# *Andersonoplatus*, a new, remarkable leaf litter inhabiting genus of Monoplatina (Coleoptera, Chrysomelidae, Galerucinae, Alticini)

**DOI:** 10.3897/zookeys.744.22766

**Published:** 2018-03-20

**Authors:** Adelita M. Linzmeier, Alexander S. Konstantinov

**Affiliations:** 1 Universidade Federal da Fronteira Sul – UFFS, Rua Edmundo Gaievski, 1000, sala 211, 85.770-000, Realeza – PR, Brazil; 2 Systematic Entomology Laboratory, USDA, Smithsonian Institution, P.O. Box 37012, National Museum of Natural History, Washington, DC 20013-7012, USA

**Keywords:** Alticini, flightless, leaf litter, Neotropical region, new genus, new species

## Abstract

*Andersonoplatus*, a new genus with 16 new species from Venezuela (*A. andersoni*, *A. bechyneorum*, *A. castaneus*, *A. flavus*, *A. jolyi*, *A. laculata*, *A. lagunanegra*, *A. macubaji*, *A. merga*, *A. merida*, *A. microoculus*, *A. peck*, *A. rosalesi*, *A. sanare*, *A. saviniae*) and Panama (*A. baru*), is described and illustrated. All the specimens were collected in leaf litter by R. Anderson and S. and J. Peck. *Andersonoplatus* is compared to *Andersonaltica* Linzmeier & Konstantinov, *Apleuraltica* Bechyne, *Distigmoptera* Blake and *Ulrica* Scherer.

## Introduction

**Figure 1.**
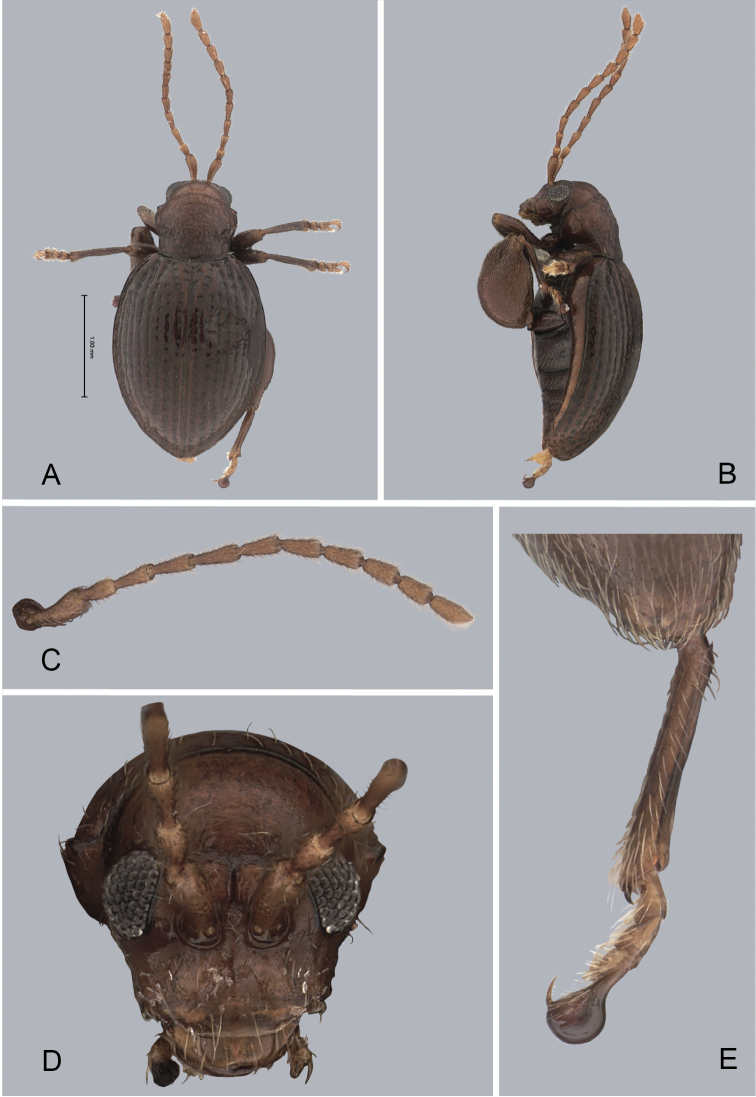
*Andersonoplatus andersoni*. **A** Habitus dorsal **B** Habitus lateral **C** Antenna **D** Head, frontal view **E** Hind leg.

The Monoplatina (Chrysomelidae, Galerucinae, Alticini) was established by Chapuis (1875) to group 42 genera described by Clark (1860). Monoplatina currently contains 47 genera and more than 560 species being mainly distributed in the Neotropical region, mostly in South America (Linzmeier and Konstantinov 2009, 2012). Monoplatina flea beetles can be diagnosed within Alticini by the globose fourth visible metatarsomere (Fig. 1E), the closed or very narrowly open procoxal cavities, and by a very thick metafemur, usually as wide as long and most of the times longer than the metatibia.

Recent collecting of leaf litter inhabiting beetles in Central America (Anderson 2010) revealed an entirely new fauna of flea beetles. The first group of this fauna was described recently (Linzmeier and Konstantinov 2012). The second group is being described below.

## Materials and methods

Most specimens described in this paper were collected by R. Anderson of the Canadian Museum of Nature as part of his long term studies of weevils (and other beetles) from leaf litter in the New World.

Dissecting techniques and terminology used follow Konstantinov (1998). Specimen observations were made with a Zeiss Stemi SV11 Apo microscope. Digital photographs were taken with Axio Zoom V16 microscope and AxioCam HRC digital camera attached to it. The holotypes will be deposited in Museo del Instituto de Zoologia, UCV, Maracay, Venezuela (**MIZA**), currently and temporarily they are in the National Museum of Natural History, Smithsonian Institution, Washington DC (**USNM**). Paratypes are split between collection of the Canadian Museum of Nature, Ottawa, Canada (**CMNC**) and USNM.

## Taxonomy

### Andersonoplatus gen. n.

Taxon classificationAnimaliaColeopteraChrysomelidae

http://zoobank.org/AEF0385C-245F-4A58-8EF3-BC1CD3E5EF5F

Figs 1 –35

#### Description.

Body length 1.62–4.00 mm, width 0.81–1.78 mm, sparsely pilose to pilose, elliptical, moderately flat to convex in lateral view. Color yellow to pale brown to dark. Apterous.

*Head*: hypognathous, flat to slightly convex in lateral view, generally smooth or reticulated, sparsely pubescent. Frons and vertex flat or forming a 135° angle in lateral view. Supraorbital pore small, almost indistinguishable to large, generally among other pores, bearing a seta. Antennal callus generally longer than wide, rounded to quadrate separated by long midfrontal sulcus, delineated from vertex by a shallow or deep, straight or inclined sulcus, entering interantennal space. Suprantennal sulcus well developed. Orbit narrow. Antennal socket elongatew or rounded. Frontal ridge short, wider at middle or V-shaped, usually poorly defined laterally. Anterofrontal ridge generally long, relatively tall, oblique, poorly defined. Eyes large to very small generally rounded. Clypeus long. Labrum slightly notched in middle, with six setiferous pores, four with log setae and two with short setae. First maxillary palpomere as wide as long, as wide as the second. Second maxillary palpomere twice as long as first, globose in some species. Third maxillary palpomere thinner, conical, and as long as the first. First labial palpomeres quadrate, second longer than first and, third smaller, thinner and conical. Antenna with eleven antennomeres, filiform to moniliform.

*Thorax*: pronotum trapezoidal, narrower than elytra, anterior margin straight, wider than posterior; posterior margin nearly straight to slightly convex; lateral margin sinuated. Anterior and posterior angle generally bearing seta, anterior angles in some species pointed outward. Surface shiny to dull, generally reticulated and with punctuation shallow and disperse to deep and well defined; pilosity short and sparse to dense. Post basal impression present, generally absent in middle, but represented by two generally shallow, rounded impressions laterally. Some species have lateral margin notched near middle. Pronotal disc flat to weakly raised. Scutellum rounded to triangular, wider than long, setose. Prosternal surface reticulated to punctuated. Prosternal intercoxal process narrow or thin, generally margined, extended posteriorly beyond coxa ending in a triangular form. Posterior end nearly twice as wide as middle. Procoxae globose. Procoxal cavities closed to narrowly open posteriorly. Mesosternum as long as prosternal process, T-shaped, straight posteriorly. Metasternum smooth, with sparse pilosity, convex in lateral view, shorter than pro- and mesosternum together; posterior margin with deep furrow medially that runs longitudinally along 1/3 of metasternum. Elytra elliptical, generally fused, truncate at apex. Elytral surface shiny, with sparse to dense semi-erect hairs. Punctures forming seven or nine striae (excluding short scutellar and marginal striae). Interspaces flat to convex. Humeral and basal calli generally absent. Epipleura wide, sinuous, nearly vertical or nearly horizontal, narrowing at elytral apex, reaching it.

Fore- and midlegs with femora slightly dilated and thickened toward apex; tibiae subcylindrical, somewhat enlarged toward apex; apex of tibiae with row of denticles; pubescence sparsely distributed. First and second pro- and mesotarsomeres similar in size, as wide as long; third tarsomere varies in length; fourth visible tarsomere as long as the first and second together. In males the first pro- and mesotarsomeres more globose. Metafemur greatly enlarged, longer than wide and longer than metatibia. Metatibia nearly straight in lateral view, curved or nearly straight in dorsal view. Outer lateral dorsal ridge ending in an apical tooth followed by numerous denticles. Inner lateral dorsal with some denticles at end, in some species ending in an apical tooth. Metatibial spur generally short. Metatarsomeres one to three variable in length, generally similar in size; third metatarsomere not bilobed; visible globose, swollen with its base elongate. Claws simple or appendiculate and long.

*Abdomen*: sparsely pubescent, reticulated, sparsely punctured, with five visible ventrites. Fifth ventrite variable in length, with distinct sexual dimorphism: males with small salient lobe located centrally on posterior margin and perpendicular line; females with last ventrite evenly conical at apex. Posterior margin of fourth ventrite straight to concave. Abdominal pleurites as sclerotized as ventrites.

*Male genitalia*: median lobe simple, convex in lateral view; in ventral view, with lateral margins almost parallel, apex subtriangular, slightly protruding into more or less differentiated denticle, in some species round on top. Basal part long and bent ventrally in lateral view.

*Female genitalia*: eighth tergite with rounded anterior margin, more sclerotized laterally, bearing many moderately long setae. Tignum long, narrow, with central canal; posterior and anterior sclerotization variable in shape. Vaginal palpi elongate, posteriorly and anteriorly strongly sclerotized, each with approximately eight setae at apex. Palpi narrowly rounded at apex, enlarged at last third but thinned at apex, situated close together and merged anteriorly for more than half of their length. Spermatheca curved, with receptacle and pump not differentiated from each other. Apex of pump with spoon-like projection. Spermathecal duct long, not forming coils.

#### Type species.

*Andersonoplatus microoculus* Linzmeier & Konstantinov, sp. n.

#### Etymology.

We dedicate this new genus to R. Anderson for his remarkable discoveries of leaf litter flea beetles in the New World. The name is masculine.

#### Differential diagnosis.

*Andersonoplatus* differs from all other known genera of Monoplatina in having the dorsoventrally flat, elliptical elytra and the pronotum being trapezoidal, usually anteriorly wider than posteriorly, much narrower than elytra with sinuate lateral margin. All the *Andersonoplatus* species are apterous with mostly fused elytra, lacking wings. Flightlessness is a common feature of leaf litter or other substrate living leaf beetles. Other apterous Monoplatina species are placed in *Andersonaltica* Linzmeier & Konstantinov, 2012, *Apleuraltica* Bechyne, 1986, *Distigmoptera* Blake, 1943 and *Ulrica* Scherer, 1962. *Andersonoplatus* can be easily differentiated from *Andersonaltica* in having mostly filiform antennae. Antennae in *Andersonaltica* are clubbed. *Andersonoplatus* can be differentiated from *Apleuraltica* and *Distigmoptera* based on having pronotum laterally margined with distinct border and mostly flat disc. In *Apleuraltica* and *Distigmoptera* the lateral margin of pronotum is lacking distinct border (or with very faint one in *Distigmoptera*) and the disc is with two noticeable bumps separated by a longitudinal impression. *Andersonoplatus* can be differentiated from *Ulrica* by a body thinner in lateral view and the pronotum being anteriorly wider than posteriorly. In *Ulrica* the body is thicker in lateral view and the pronotum being anteriorly narrower than posteriorly.

### Andersonoplatus andersoni sp. n.

Taxon classificationAnimaliaColeopteraChrysomelidae

http://zoobank.org/0603250A-C52E-4017-BF6E-85E506F3E4AD

Figs 1 , 2

#### Description.

Body length 2.43–3.02 mm, width 1.24–1.72 mm, pronotum and elytra with sparse, semi-erect hairs, shiny, moderately convex in lateral view. Color pale brown to dark brown.

*Head* (Fig. 1D): slightly convex in lateral view, generally smooth with fine reticulation and few punctures of different size and shape above antennal callus, gena shiny, with few sparse punctures and sparse pilosity. Frons and vertex forming almost a 135° angle in lateral view. Antennal callus delineate from vertex by deep and straight supracallinal sulcus. Antennal callus elevated above vertex; surface even, with no or two punctures, if bearing setae, they are short. Orbital sulcus deep. Supraorbital sulcus represented by few deep punctures near antennal socket, absent near supracallinal sulcus. Supraorbital and supracallinal sulci not connected. Suprafrontal sulcus shallow. Frontolateral sulcus deep. Frontogenal suture deep. Frontal ridge short and narrow, widest in middle. Anterofrontal ridge long, relatively tall, oblique. Antennae filiform. The last five antennomeres slightly wider and shorter than antennomeres III-VI; antennomere II shortest.

*Thorax*: pronotum (Fig. 1A, B) much narrower than elytra, notched laterally near middle. Anterior margin straight, wider than posterior, posterior margin nearly straight, lateral margin slightly sinuated. Surface reticulated, granulated, with very short and sparse pilosity and two vague impressions below middle, sparsely covered with variously defined punctures, diameter of which smaller than distance between punctures. Prosternal surface densely punctate. Elytra fused. Elytral surface shiny, with sparse, white, semi-erect hairs. Punctures (Fig. 1A) forming nine striae, ninth stria merge with marginal one. Interspaces convex. Punctures at base of fifth and sixth striae deeper than other having fold-like appearance. Second and third striae not reaching elytral base. Epipleura nearly vertical. Metafemur longer than wide and 1.46 times longer than metatibia. Claws appendiculate and long.

**Figure 2.**
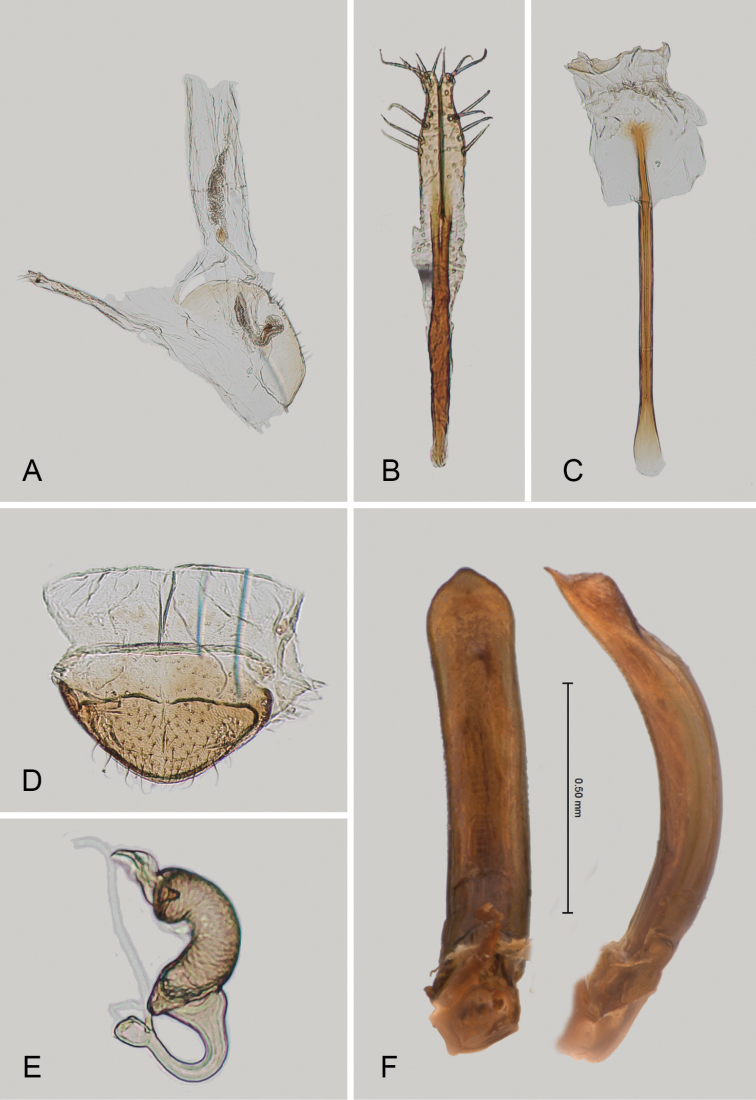
*Andersonoplatus andersoni*. **A** Female genitalia, lateral view **B** Vaginal palpi **C** Tignum **D** Last abdominal tergite of female **E** Spermatheca **F** Median lobe of aedeagus, ventral and lateral views.

*Male genitalia* (Fig. 2F): median lobe simple, convex in lateral view; in ventral view, with lateral margins lightly concave, apex subtriangular, slightly protruding, and rounded on top. Ventral side with shallow longitudinal impression bottom of which covered with transverse wrinkles, sides of impression not forming ridges. Basal part long and bent ventrally in lateral view.

*Female genitalia* (Fig. 2A–E): eighth tergite with rounded posterior margin, more sclerotized laterally, bearing many moderately long setae (Fig. 2D). Tignum long, narrow, with central canal; posterior area broad, truncate; anterior area spatulate (Fig. 2C). Vaginal palpi elongate, basally strongly sclerotized, each with approximately eight setae at apex (Fig. 2B). Palpi rounded at apex, enlarged at last third but thinned at apex, situated close together and merged anteriorly for more than half of their length. Spermatheca curved, with receptacle and pump not differentiated from each other. Apex of pump with spoon-like projection. Spermathecal duct long, widest at base, without coils, making relatively long loop (Fig. 2E).

#### Type material.

**Holotype**, ♂. VENEZUELA: Trujillo/ camino viejo a Trujillo, Paramo/ La Cristalina, km 9.7, 2400m/ 09°21'21"N, 70°17'51"W/ 20.V.1998-022F/ R.Anderson, elfin for. Litter (MIZA). **Paratypes** (7♂ 4♀ USNM). (2♂ USNM) same label as holotype except: (1♂ USNM) “022A”; (3♂1♀ CMNC) “022E”; (1♂3♀ USNM) “022J”.

#### Etymology.

We name this species after R. Anderson. Regardless how many taxa we name after him, his remarkable discoveries of leaf litter flea beetles in the New World would warrant many more.

#### Differential diagnosis.

*Andersonoplatus andersoni* is similar to *A. sanare* but can be differentiated from it based on the following characters: ventral side of median lobe with shallow longitudinal impression bottom of which covered with transverse wrinkles (Fig. 2F) and spermathecal duct making relatively long loop (Fig. 2E). In *A. sanare*: ventral side of median lobe without longitudinal impression (Fig. 33A) and spermathecal duct making relatively short loop (Fig. 33E).

### Andersonoplatus baru sp. n.

Taxon classificationAnimaliaColeopteraChrysomelidae

http://zoobank.org/41C61682-A5A2-4231-9DF7-96D4BDA2F57D

Figs 3 , 4

#### Description.

Body length 3.39–3.40 mm, width 1.62–1.67 mm, moderately shiny, densely pilose, with semi-erect hairs, flat in lateral view. Uniform yellow with antennae and legs slightly lighter than body.

**Figure 3.**
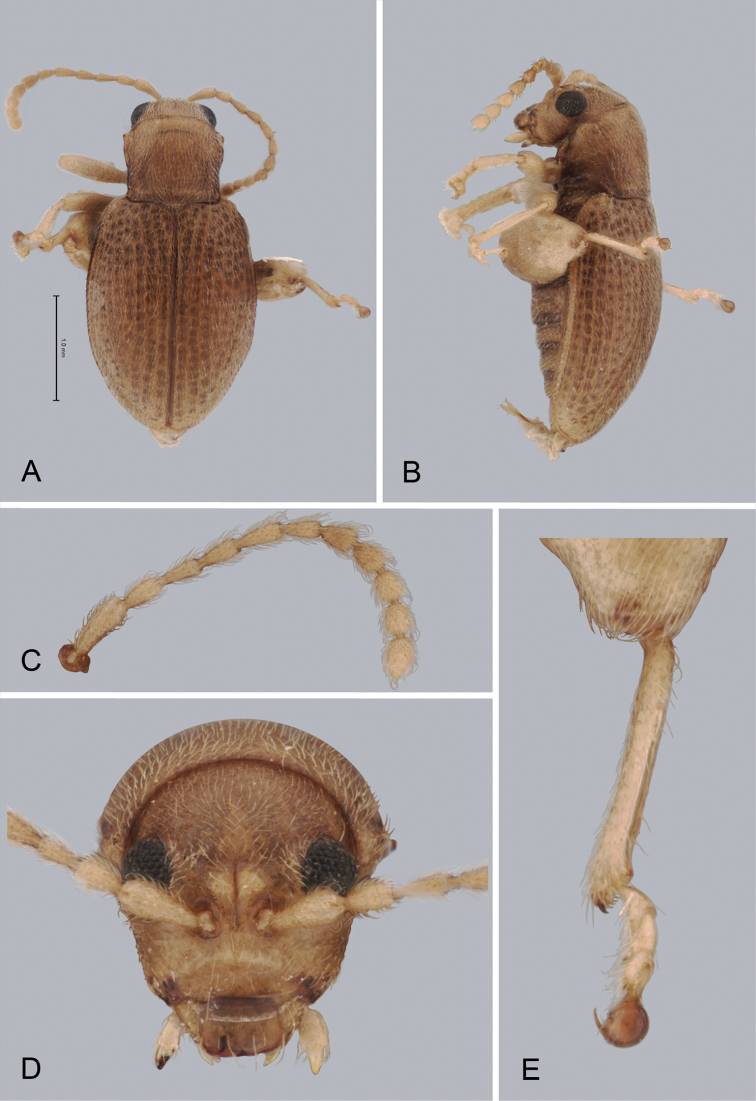
*Andersonoplatus baru*. **A** Habitus dorsal **B** Habitus lateral **C** Antenna **D** Head, frontal view **E** Hind leg.

*Head* (Fig. 3B, D): slightly convex in lateral view, moderately shiny, generally reticulated, and densely pilose. Frons and vertex forming near a 135° angle in lateral view. Antennal callus delimited from vertex by straight sulcus; slightly elevated above vertex; surface uneven, with more than two punctures, some of them bearing setae. Orbital sulcus shallow. Supraorbital sulcus deep not connected with supracallinal. Suprafrontal and frontolateral sulcus absent. Frontogenal suture shallow. Orbit narrower than transverse diameter of antennal socket. Interantennal space narrower than transverse diameter of eye and as wide as transverse diameter of antennal socket. Frontal ridge short and narrow. Anterofrontal ridge short, relatively tall, oblique. Last five antennomeres shorter and wider than second.

*Thorax*: pronotum (Fig. 3A, B) slightly trapezoidal, narrower than elytra. Anterior margin wider than the posterior, posterior margin straight, lateral margin slightly sinuated. Surface reticulated, densely punctate, densely pilose. Pronotal disc dull. Scutellum triangular, wider than long, reticulated. Prosternal surface reticulated. Posterior end nearly twice as wide as middle. Elytra fused. Elytral surface dull, pilose, with semi-erect hairs, deeply punctate (Fig. 3A). Punctures forming nine striae. Interspaces convex, with small punctures. Marginal elytral stria consisting of two punctures. Second and third striae reaching elytral base. Epipleura nearly vertical. Metafemur longer than wide and 1.57 times longer than metatibia. Metatibia almost straight in lateral view, slightly curved in dorsal view. Claws simple and long. Posterior margin of fourth ventrite nearly straight. Males unknown.

**Figure 4.**
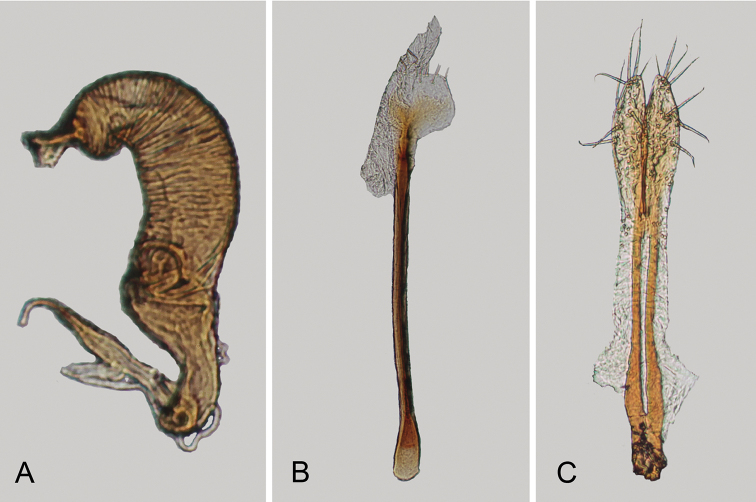
*Andersonoplatus baru*. **A** Spermatheca **B** Tignum **C** Vaginal palpi.

*Female genitalia* (Fig. 4A–C): tignum long, narrow, with central canal; posterior area broad, sclerotization poorly delineated; anterior area spatulate (Fig. 4B). Vaginal palpi elongate, basally strongly sclerotized, each with approximately eight setae at apex (Fig. 4C). Palpi rounded at apex, enlarged at last third but thinned at apex, situated close together and merged anteriorly for more than half of their length. Posterior sclerotization of vaginal palpi with convex sides. Spermatheca curved, with receptacle and pump not differentiated from each other. Apex of pump with spoon-like projection. Spermathecal duct long, widest at base, without coils (Fig. 4A).

#### Type material.

**Holotype**, ♀. PANAMA: Chiriquí/ P.Nac. Volcan Baru, 5.9/ km E. Cerro Punta, 2400m/ 14.VI.1995-21B, R.S. Ander-/ son, oak ridge bamboo for. litt. (MIZA). **Paratype** (1♀ USNM). Same label as holotype except “21G”.

#### Etymology.

This species is a noun in apposition based on the type locality, volcano Baru in Chiriqui mountains where it was collected.

#### Diagnosis.

Dorsal surface densely covered with hairs, light straw color, second and third elytral striae reaching elytral base.

### Andersonoplatus bechyneorum sp. n.

Taxon classificationAnimaliaColeopteraChrysomelidae

http://zoobank.org/A103349B-1412-4E92-AF34-96FEC964836C

Figs 5 , 6

#### Description.

Body length 2.32–2.64 mm, width 1.29–1.40 mm, pronotum and elytra with sparse, semi-erect hairs, shiny, elliptical, moderately convex in lateral view. Color castaneous.

**Figure 5.**
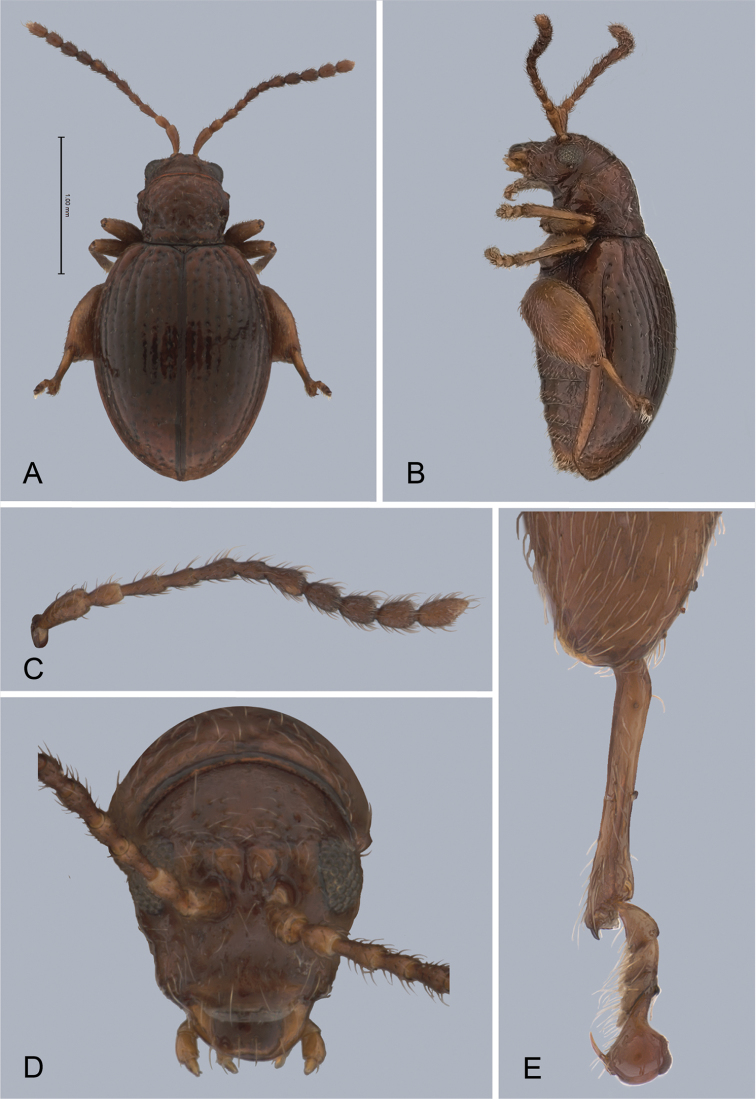
*Andersonoplatus bechyneorum*. **A** Habitus dorsal **B** Habitus lateral **C** Antenna **D** Head, frontal view **E** Hind leg.

*Head* (Fig. 5B, D): slightly convex in lateral view, generally smooth, gena and frons shiny with sparse pilosity. Frons and vertex forming an angle of approximately 135° in lateral view. Antennal callus delineate from vertex by shallow and straight supracallinal sulcus. Antennal callus slightly elevated above vertex, surface even, with no or two punctures, if bearing setae, they are short. Orbital sulcus shallow. Supraorbital sulcus represented by few deep punctures near antennal socket, absent near supracallinal sulcus. Supraorbital and supracallinal sulcus not connected. Suprafrontal sulcus absent. Frontolateral sulcus shallow. Frontogenal suture indistinguishable. Interantennal space narrower than transverse diameter of eye and as wide as transverse diameter of antennal socket. Frontal ridge short and narrow. Last five antennomeres slightly wider than preceding ones.

*Thorax*: pronotum (Fig. 5A, B) much narrower than elytra, notched laterally below middle. Anterior margin straight, wider than posterior, posterior margin nearly straight, lateral margin sinuated. Anterior angles acute. Surface deeply granulate, with pilosity very short and very sparse. Pronotal disc weakly raised. Post basal impression present, with deeper rounded impressions laterally. Scutellum rounded, much shorter than wide, setose. Prosternal surface reticulated. Posterior end of intercoxal process nearly twice as wide as middle. Elytra fused. Elytral surface shiny, with very sparse, white, semi-erect hairs, deeply punctated (Fig. 5A). Punctures forming nine striae, the ninth stria overlapping with marginal one. Each punctation bears one very short setae (some setae can be found on the interspaces). Interspaces very convex. Punctures at base of fifth and sixth striae deeper than other having fold-like appearance. Marginal line of elytra interrupted at base. Second and third striae not reaching elytral base. Epipleura slightly convex, nearly vertical. Metafemur longer than wide and 1.60 times longer than metatibia. Claws simple and long.

**Figure 6.**
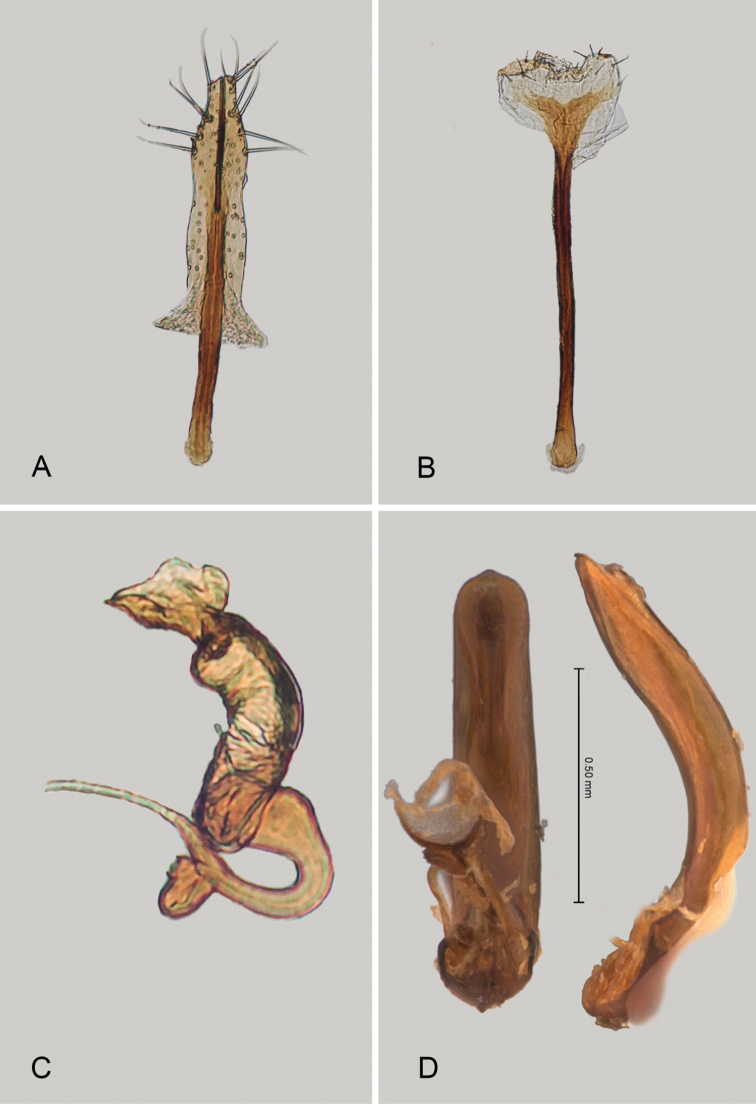
*Andersonoplatus bechyneorum*. **A** Vaginal palpi **B** Tignum **C** Spermatheca **D** Median lobe of aedeagus, ventral and lateral views.

*Male genitalia* (Fig. 6D): ventral side with deep longitudinal impression with bottom lacking transverse wrinkles, sides of impression form high ridges. Apical denticle sharply bent ventrally.

*Female genitalia* (Fig. 6A–C): tignum long, narrow, with central canal; posterior area broad, sclerotization relatively well delineated; anterior area spatulate (Fig. 6B). Vaginal palpi elongate, basally strongly sclerotized, each with approximately eight setae at apex (Fig. 6A). Palpi pointed at apex, enlarged at last third but thinned at apex, situated close together and merged anteriorly for more than half of their length. Spermatheca curved, with receptacle and pump not differentiated from each other. Apex of pump with spoon-like projection. Spermathecal duct short, widest at base, without coils (Fig. 6C).

#### Type material.

**Holotype**, ♂. VENEZUELA: Trujillo/ camino viejo a Trujillo, Paramo/ La Cristalina, km 9.7, 2400m/ 09°21'21"N, 70°17'51"W/20.V.1998-022B/ R.Anderson, elfin for. litter (MIZA). **Paratypes** (5♂ USNM, 1♀ CMNC). Same label as holotype.

#### Etymology.

We name this species after Mila and Jan Bechyne who together made large contribution to our knowledge of mostly Neotropical leaf beetles describing 143 genera and 2290 species.

#### Differential diagnosis.

*Andersonoplatus bechyneorum* can be differentiated from most *Andersonoplatus* species by the following characters: pronotal surface uneven, covered with relatively large but poorly defined punctures (Fig. 5A) and median lobe of aedeagus ventrally with two ridges and deep grove between them (Fig. 6D).

### Andersonoplatus castaneus sp. n.

Taxon classificationAnimaliaColeopteraChrysomelidae

http://zoobank.org/49729921-C29A-4A50-B370-623232423114

Figs 7 , 8 , 9

#### Description.

Body length 2.59–3.29 mm, width 1.45–1.78 mm, pronotum and elytra with very sparse, semi-erect hair, shiny, moderately convex in lateral view. Color brown to chestnut brown with a pearl luster; antennae and legs much lighter.

**Figure 7.**
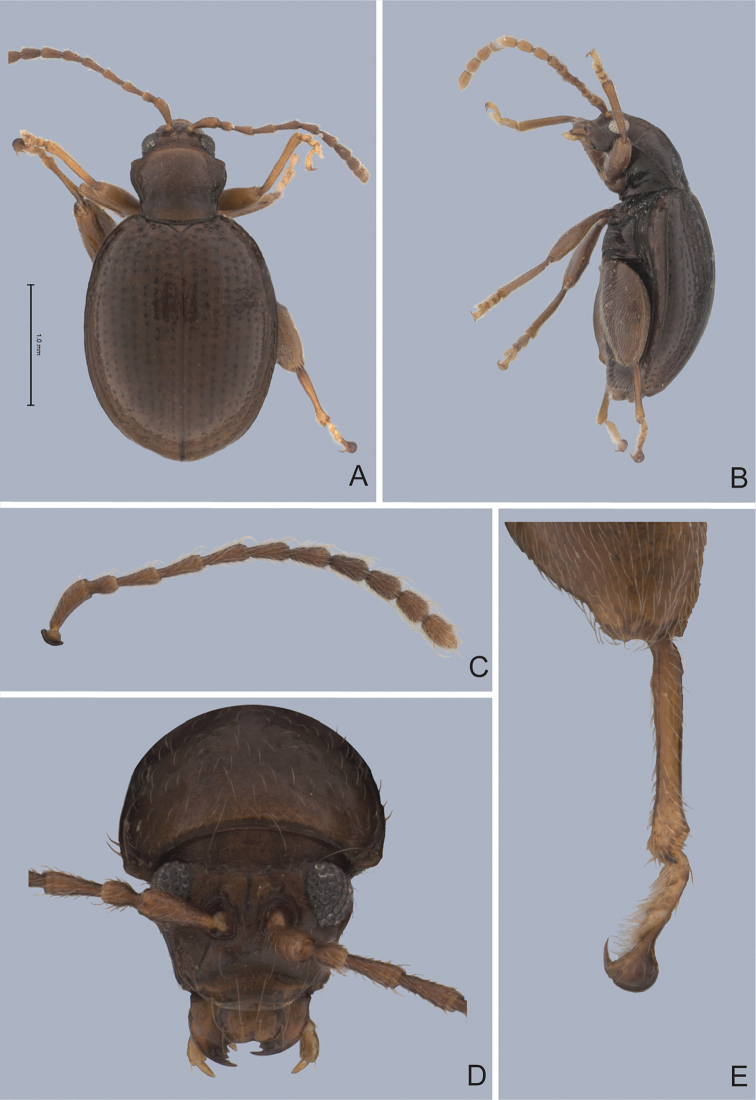
*Andersonoplatus castaneus*. **A** Habitus dorsal **B** Habitus lateral **C** Antenna **D** Head, frontal view **E** Hind leg.

*Head* (Fig. 7B, D): slightly convex in lateral view, generally smooth with fine reticulation, gena with sparse pilosity. Frons and vertex forming nearly a 135° angle in lateral view. Antennal callus delimited from vertex by deep and slightly inclined supracallinal sulcus. Antennal callus raised, surface even, with no or two punctures, if bearing setae, they are short. Orbital sulcus deep. Supraorbital sulcus absent. Suprantennal sulcus deep. Suprafrontal sulcus absent. Frontolateral sulcus shallow. Frontogenal suture well developed. Subgenal suture well developed along base of mandible. Orbit narrower than transverse diameter of antennal socket. Interantennal space narrower than transverse diameter of eye and as wide as transverse diameter of antennal socket. Frontal ridge short and narrow. Eyes with nearly more than 20, small ommatidia. The last five antennomeres as long as sixth, slightly wider than preceding ones; second antennomere shortest (Fig. 7C).

*Thorax*: pronotum (Fig. 7A, B) much narrower than elytra, notched laterally below middle. Anterior margin wider than posterior, posterior margin slightly concave, lateral margin sinuated. Anterior angles pointed outward. Surface reticulated, with pilosity very short and sparse, lacking punctures. Pronotal disc weakly raised. Scutellum triangular. Prosternal surface reticulated. Prosternal intercoxal process narrow. Posterior end twice as wide as middle. Elytra weakly fused. Elytral surface shiny, with very sparse, white, semi-erect hairs, and a pearl luster. Punctures (Fig. 7A) forming nine striae (marginal stria consisting of one or two punctures). Elytral interspaces flat. Second and third striae reaching elytral base. Epipleura nearly vertical. Metafemur greatly enlarged, longer than wide and 1.76 times longer than metatibia. Claws simple and long.

**Figure 8.**
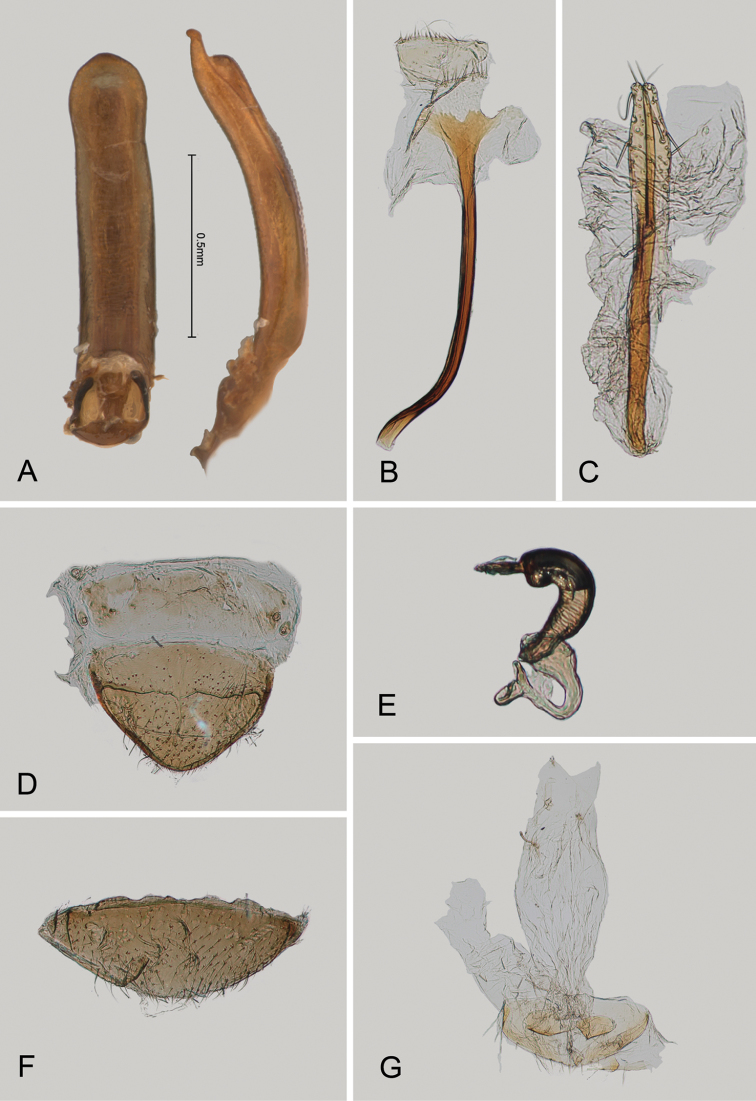
*Andersonoplatus castaneus*. **A** Median lobe of aedeagus, ventral and lateral views **B** Tignum **C** Vaginal palpi **D** Last abdominal tergite of female **E** Spermatheca **F** Last abdominal sternite of female **G** Female genitalia, ventral view.

*Male genitalia* (Fig. 8A): ventral side with longitudinal impression with bottom lacking transverse wrinkles, sides of impression form ridges. Apical denticle not developed in ventral view, apex bent ventrally.

**Figure 9.**
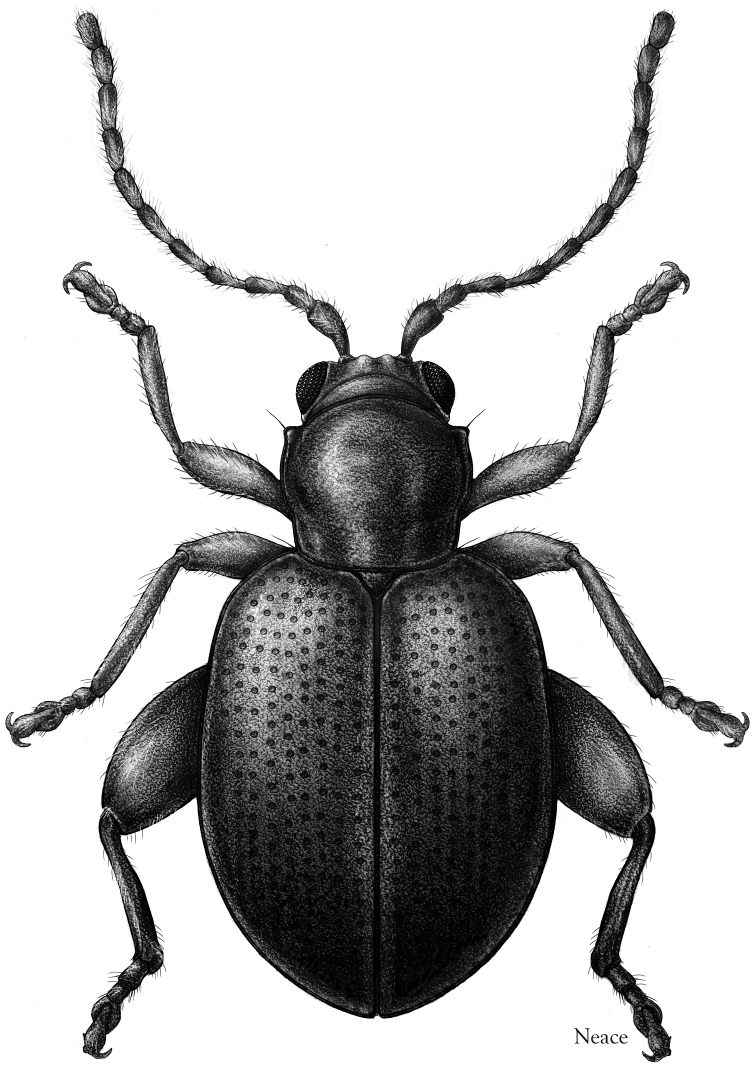
*Andersonoplatus castaneus*. Dorsal habitus, illustration by Meghan Neace.

*Female genitalia* (Fig. 8B–G): tignum long, narrow, bent, with central canal; posterior area broad, sclerotization relatively well delineated; anterior area weakly widened (Fig. 8B). Vaginal palpi elongate, basally strongly sclerotized, each with approximately eight setae at apex (Fig. 8C). Palpi pointed at apex, enlarged at last third but thinned at apex, situated close together and merged anteriorly for more than half of their length. Spermatheca curved, with receptacle and pump not differentiated from each other. Apex of pump with spoon-like projection. Spermathecal duct short, widest at base, without coils (Fig. 8E).

#### Type material.

**Holotype**, ♂. VENEZUELA: Trujillo/ camino Viejo a Trujillo, Paramo/ La Cristalina, km 9.7, 2400m/ 09°21'21"N, 70°17'51"W/ 20.V.1998-022C (MIZA). **Paratypes** (6♂ 5♀ USNM). Same label as holotype except: (1♂1♀ CMNC) “022D”; (1♂1♀ USNM) “022F”; (3♂2♀ USNM) “022J”; (1♀ CMNC) “022E”; (1♂ CMNC) “camino viejo a Trujillo/ km 6.0, 2240m/ 09°21'03"N, 70°17'36"W/ E.Anderson, cloud for. litter”.

#### Etymology.

The specific epithet is a noun in apposition based on the color of the beetles.

#### Differential diagnosis.

*Andersonoplatus castaneus* is similar to *A. jolyi* and can be differentiated from it based on the following characters: supracallinal sulci well developed, deep (Fig. 7D); apex of median lobe of aedeagus bent ventrally in lateral view (Fig. 8A).

### Andersonoplatus flavus sp. n.

Taxon classificationAnimaliaColeopteraChrysomelidae

http://zoobank.org/2361E43C-6838-4339-A589-128B453FAA84

Figs 10 , 11

#### Description.

Body length 2.70–2.91 mm, width 1.40–1.51 mm, pronotum and elytra with sparse, semi-erect hairs, shiny, moderately convex in lateral view. Color yellow.

**Figure 10.**
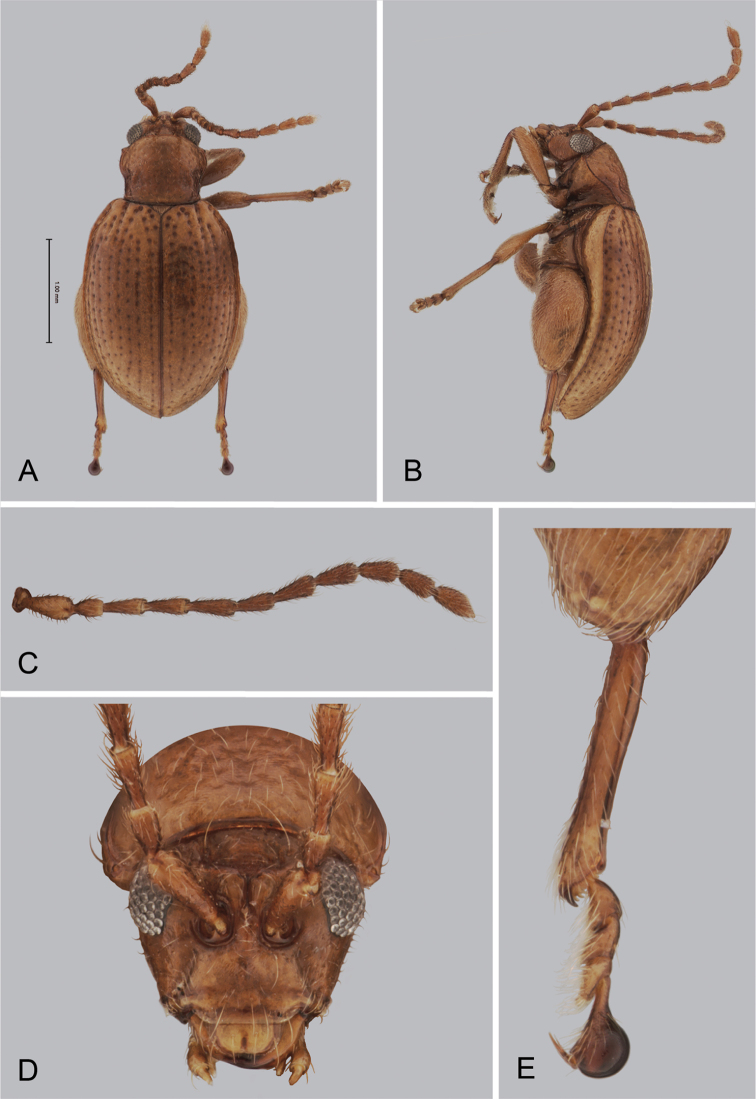
*Andersonoplatus flavus*. **A** Habitus dorsal **B** Habitus lateral **C** Antenna **D** Head, frontal view **E** Hind leg.

*Head* (Fig. 10B, D): slightly convex in lateral view, vertex smooth with a fine reticulation, gena shiny, slightly punctuated with sparse pilosity. Frons and vertex forming nearly a 135° angle in lateral view. Antennal callus delineated from vertex by deep and inclined supracallinal sulcus. Antennal callus elevated above vertex, surface even, with no or two punctures, if bearing setae, they are short. Orbital sulcus deep. Supraorbital sulcus shallow, almost connected with supracallinal sulcus. Suprafrontal and frontolateral sulci deep. Frontogenal suture well developed. Orbit narrower than transverse diameter of antennal socket. Interantennal space narrower than transverse diameter of eye and transverse diameter of antennal socket separately. Frontal ridge short and narrow. Antennae filiform; antennomeres three to eleven similar in length with last five ones slightly wider; second antennomere shortest (Fig. 10C).

*Thorax*: pronotum (Fig. 10A) much narrower than elytra, notched laterally nearly at middle. Anterior margin wider than posterior, posterior margin nearly straight, lateral margin slightly sinuated. Anterior angles pointed outward. Surface reticulated, sparsely covered with large punctures, with very short and very sparse hairs, sparsely covered with variously defined punctures, diameter of which smaller than distance between punctures. Pronotal disc weakly raised. Scutellum triangular, much shorter than wide. Prosternal surface reticulated. Prosternal intercoxal process narrow. Posterior end twice as wide as middle. Elytra fused. Elytral surface shiny, with sparse, white, semi-erect hairs. Punctures forming nine striae, ninth stria merge with marginal one. Interspaces slightly convex. Punctures at base of fifth and sixth striae deeper than others. Second and third striae not reaching elytral base. Epipleura nearly vertical, with a line of punctation along internal margin. Metafemur greatly enlarged, 1.59 times longer than metatibia. Claws appendiculate, long.

**Figure 11.**
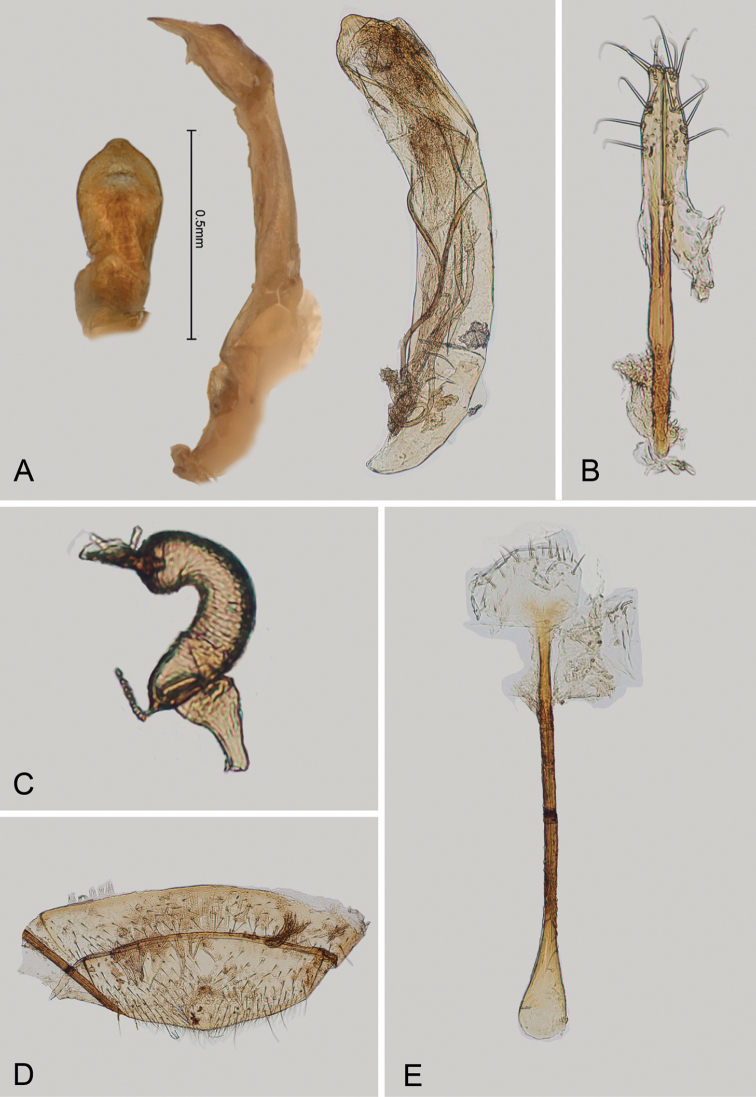
*Andersonoplatus flavus*. **A** Median lobe of aedeagus, ventral, lateral views, internal structures under compound scope **B** Vaginal palpi **C** Spermatheca **D** Last abdominal sternite of female **E** Tignum.

*Male genitalia* (Fig. 11A): apical denticle well developed, wide in ventral view, apex straight, not bent ventrally.

*Female genitalia* (Fig. 11B–E): tignum long, narrow, slightly bent, with central canal; posterior area broad, sclerotization poorly delineated, anterior area weakly spatulate (Fig. 11E). Vaginal palpi elongate, basally strongly sclerotized, each with approximately eight setae at apex (Fig. 11B). Palpi pointed at apex, enlarged at last third but thinned at apex, situated close together and merged anteriorly for more than half of their length. Spermatheca curved, with receptacle and pump not differentiated from each other. Apex of pump with spoon-like projection. Spermathecal duct short, widest at base, without coils (Fig. 11C).

#### Type material.

**Holotype**, ♂. VENEZUELA: Trujillo/ camino viejo a Trujillo, Paramo/ La Cristalina, km 9.7, 2400m/ 09°21'21"N, 70°17'51"W/ 20.V.1998-022F/ R.Anderson, elfin for. litter (MIZA). **Paratypes** (1♂ 1♀ USNM) same label as holotype. (1♀ CMNC) same label as holotype data except: “022C” and “22E”.

#### Etymology.

The specific epithet is a noun in apposition based on the color of the beetles.

#### Differential diagnosis.

*Andersonoplatus flavus* can be differentiated from most *Andersonoplatus* species based on the following characters: body color yellow; pronotal surface sparsely covered with variously defined punctures, diameter of which smaller than distance between punctures; second elytral stria not reaching base; supracallinal sulci very deep; antennomeres longer than in most species of genus.

### Andersonoplatus jolyi sp. n.

Taxon classificationAnimaliaColeopteraChrysomelidae

http://zoobank.org/D60F429D-7A7E-4EAA-B9B2-1E33156800D1

Figs 12 , 13

#### Description.

Body length 2.59–2.97 mm, width 1.29–1.40 mm, shiny, pilose, slightly flat in lateral view. Color light brown to dark brown.

**Figure 12.**
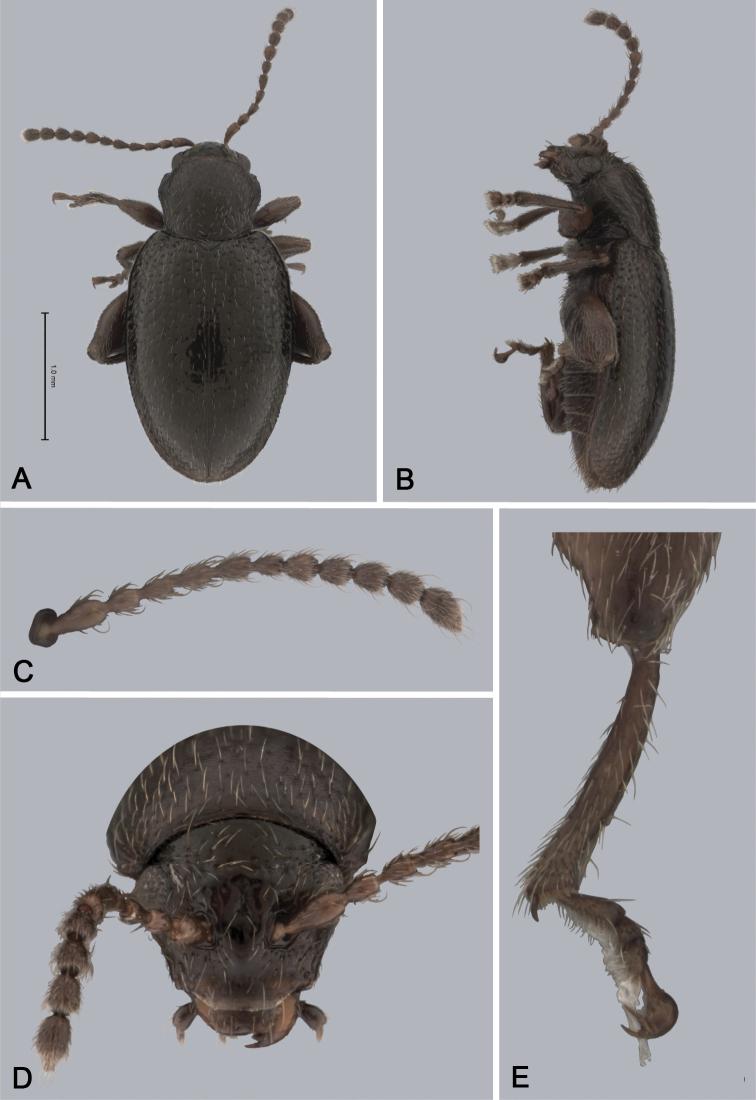
*Andersonoplatus jolyi*. **A** Habitus dorsal **B** Habitus lateral **C** Antenna **D** Head, frontal view **E** Hind leg.

*Head* (Fig. 12B, D): slightly convex in lateral view, generally reticulate, pilose. Supracallinal sulci poorly developed, barely perceptible, or marked with few punctures. Antennal callus not raised entering interantennal space, surface even, with no or two punctures, if bearing setae, they are short. Orbital sulcus shallow, represented by a line of punctures. Supraorbital sulcus absent. Suprafrontal sulcus deep. Frontolateral sulcus absent. Frontogenal suture shallow. Orbit narrow, punctated. Interantennal space slightly wider than transverse diameter of eye and twice as wide as transverse diameter of antennal socket. Frontal ridge wide and short. Anterofrontal ridge short and shallow. Eyes with more than 20, small ommatidia. Antenna (Fig. 12C) with antennomere two similar in length to three, the last five ones moniliform, with denser setae.

*Thorax*: pronotum (Fig. 12A, B) narrower than elytra. Anterior margin, wider than posterior; posterior margin nearly straight, lateral margin sinuated. Surface reticulate, punctuate, with sparse, well visible pilosity. Pronotal disc not raised. Scutellum rounded, much shorter than wide. Prosternal surface reticulated. Prosternal intercoxal process thin. Posterior end nearly twice as wide as middle. Procoxae very close to each other. Elytra fused. Elytral surface shiny, with short, white, semi-erect hairs. Punctures (Fig. 12A) forming nine slightly confused lines. Each puncture bears one very short setae. Interspaces flat. Epipleura nearly horizontal. Metafemur elongated, 1.59 times longer than metatibia. Metatibia slightly curved in lateral and dorsal view. Outer and inner lateral dorsal ridge ending in an apical tooth followed by numerous denticles (Fig. 12E). Metatibial spur thin and long. First metatarsomere almost as long as second and third together, second and third as wide as long. Claws appendiculate and long. Fifth ventrite longer than three preceding ones.

**Figure 13.**
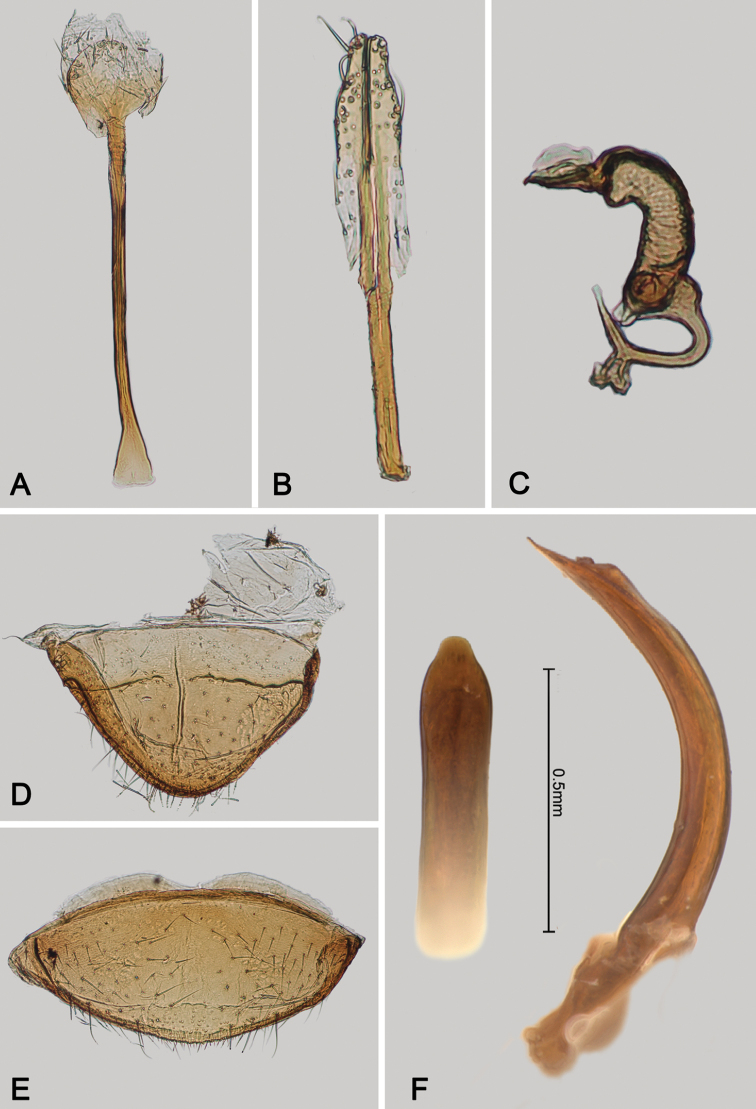
*Andersonoplatus jolyi*. **A** Tignum **B** Vaginal palpi **C** Spermatheca **D** Last abdominal tergite of female **E** Last abdominal sternite of female **F** Median lobe of aedeagus, ventral and lateral views.

*Male genitalia* (Fig. 13F): ventral side convex, shiny, with few shallow transverse wrinkles; apical denticle wide in ventral view, apex straight, not bent ventrally.

*Female genitalia* (Fig. 13A–E): tignum long, narrow, slightly bent, with central canal; anterior sclerotization widening abruptly with straight sides and apex, posterior sclerotization poorly delineated, wide, wider than anterior (Fig. 13A). Vaginal palpi elongate, basally strongly sclerotized, each with approximately eight setae at apex (Fig. 13B). Palpi pointed at apex, enlarged at last third but thinned at apex, situated close together and merged anteriorly for more than half of their length. Spermatheca curved, with receptacle and pump not differentiated from each other. Apex of pump with spoon-like projection. Spermathecal duct short, widest at base, without coils (Fig. 13C). Last abdominal sternite and tergite (Fig. 13D, E) evenly sclerotized with evenly placed setae.

#### Type material.

**Holotype**, ♂. (1) VENEZUELA: Merida/ Paseo de Aguila, Paramo de/ Mucuchies, 3740m/ 08°50'58"N, 70°48'34"W/ 21.V.1998-025B, R.Anderson. (2) dead leaves under *Espeletia timotensis* (MIZA). **Paratypes** (3♂ 7♀ USNM). (1♂2♀ USNM) same label as holotype except: (1♂1♀ CMNC) “025C”. (1♂4♀ USNM) VENEZUELA: Merida/ Alto de Timotes, Paramo/ de Mucuchies, 4000m/ 08°51'24"N, 70°49'30"W/ 26.V.1998-042, R.Anderson.

#### Etymology.

We name this species after Luis Jose Joly of Museo del Instituto de Zoologia, UCV, Maracay, Venezuela, a fellow coleopterist who contributed greatly to our knowledge of beetles of Venezuela.

#### Differential diagnosis.

*Andersonoplatus jolyi* is similar to *A. castaneus* and can be differentiated from it based on the following characters: more elongated and flat body (Fig. 12A, B); supracallinal sulci poorly developed, barely perceptible (Fig. 12D); last five antennomeres moniliform (Fig. 12C); apex of median lobe of aedeagus straight in lateral view (Fig. 13F).

### Andersonoplatus laculata sp. n.

Taxon classificationAnimaliaColeopteraChrysomelidae

http://zoobank.org/F7DBE819-E3F6-4B47-B47D-4437B68E330F

Fig. 14

#### Description.

Body length 3.89–4.00 mm, width 1.72–1.78 mm, shiny, glossy, with very sparse semi-erect hairs, almost flat in lateral view. Color black; fore- and middle legs and antennae yellow.

**Figure 14.**
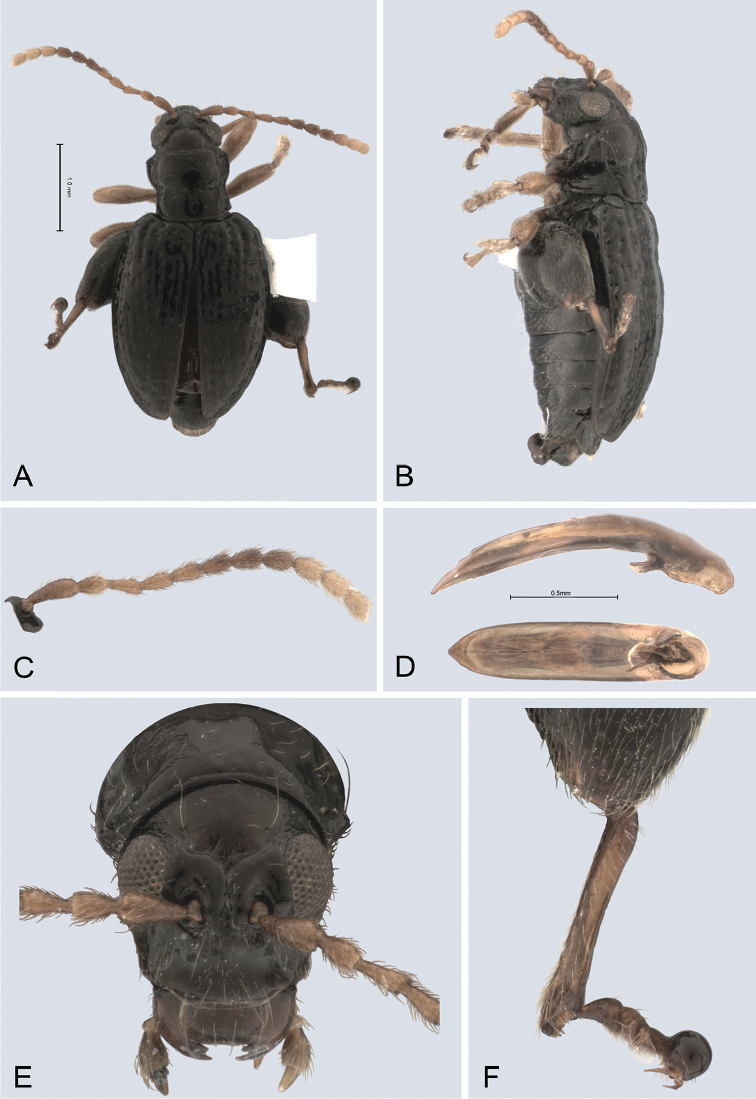
*Andersonoplatus laculata*. **A** Habitus dorsal **B** Habitus lateral **C** Antenna **D** Median lobe of aedeagus, ventral and lateral views **E** Head, frontal view **F** Hind leg.

*Head* (Figs 14A, B, E): slightly convex in lateral view, shiny, generally smooth, with very short hairs. Gena reticulated, punctuated and with sparse pilosity. Frons and vertex forming nearly a 135° angle in lateral view. Supraorbital pore small bearing a seta. Antennal callus delimited from vertex by deep and straight supracallinal sulcus, surface even, with no or two punctures, if bearing setae, they are short. Midfrontal sulcus runs from supracallinal sulcus to anterior margin of antennae. Antennal callus slightly raised. Orbital sulcus deep. Supraorbital sulcus deep, not connected with orbital sulcus. Suprafrontal and frontolateral sulcus absent. Frontogenal suture well developed. Orbit as wide as transverse diameter of antennal socket. Interantennal space narrower than transverse diameter of eye and wider than transverse diameter of antennal socket. Antennal socket rounded. Frontal ridge poorly defined, short, antennal calli nearly touching anterofrontal ridge. Anterofrontal ridge long, relatively tall, oblique. First maxillary palpomere longer than wide, shorter than second. Second maxillary palpomere slightly longer than first, globose. Antenna filiform; last six antennomeres slightly shorter and wider than three preceding ones with last three ones light in color.

*Thorax*: pronotum (Fig. 14A, B) much narrower than elytra, deeply notched at middle. Anterior margin slightly sinuated, wider than posterior, posterior margin slightly convex, lateral margin deeply sinuated. Surface smooth, glossy, with pilosity very short and sparse. Post basal impression represented by three round, shallow impressions, one longitudinally elongated medially and two laterally. Pronotal disc raised. Scutellum triangular, reticulated, wider than long. Prosternal surface reticulated and punctuated. Prosternal intercoxal process as wide as prosternum. Posterior end twice as wide as middle. Procoxal cavity narrowly open. Mesosternum reticulate, punctuate. Elytra not fused. Elytral surface shiny, glossy, with very sparse and short semi-erected hairs, deeply punctate. Punctures forming nine striae, slightly confused. Interspaces slightly convex. Humeral and basal calli shallow. Post basal impression present behind basal callus. Second stria reaching elytral base, third stria missing few punctures before elytral base. Epipleura nearly vertical, slightly narrowed at elytral apex. Metafemur 1.84 times longer than metatibia. Metatibia almost straight in lateral view, curved in dorsal view. Metatarsomeres one and two of similar size, slightly longer than third. Claws simple and long. Ventrites of nearly same length.

*Male genitalia* (Fig. 14D): ventral side flat with low longitudinal ridge apically; apical denticle poorly developed, apex straight except extreme tip that faces ventrally. Females unknown.

#### Type material.

**Holotype**, ♂. VENEZUELA: Merida/ Paramo La Culata/ 18.5km N.E. Merida, 2950m/ 08°44'34"N, 71°03'44"W/ 25.V.1998-037C, R. Anderson/ paramo, streamside shrub litter (MIZA). **Paratypes** (2♂). Same label as holotype, except (1) “037F” (USNM), (1) “037A” (CMNC).

#### Etymology.

The specific epithet is a noun in apposition based on the type locality.

#### Differential diagnosis.

*Andersonoplatus laculata* can be differentiated from most *Andersonoplatus* species based on the following characters: pronotal surface shiny, lacking punctures (Figs 14A, B, E); ventral side of aedeagus flat with low longitudinal ridge apically (Fig. 14D).

### Andersonoplatus lagunanegra sp. n.

Taxon classificationAnimaliaColeopteraChrysomelidae

http://zoobank.org/08AE5CD0-D886-4D78-BECB-960EFB4C917B

Figs 15 , 16

#### Description.

Body length 2.16–2.32 mm, width 0.97–1.02 mm, shiny, pilose, with semi-erect hairs, flat in lateral view. Color brown to dark.

**Figure 15.**
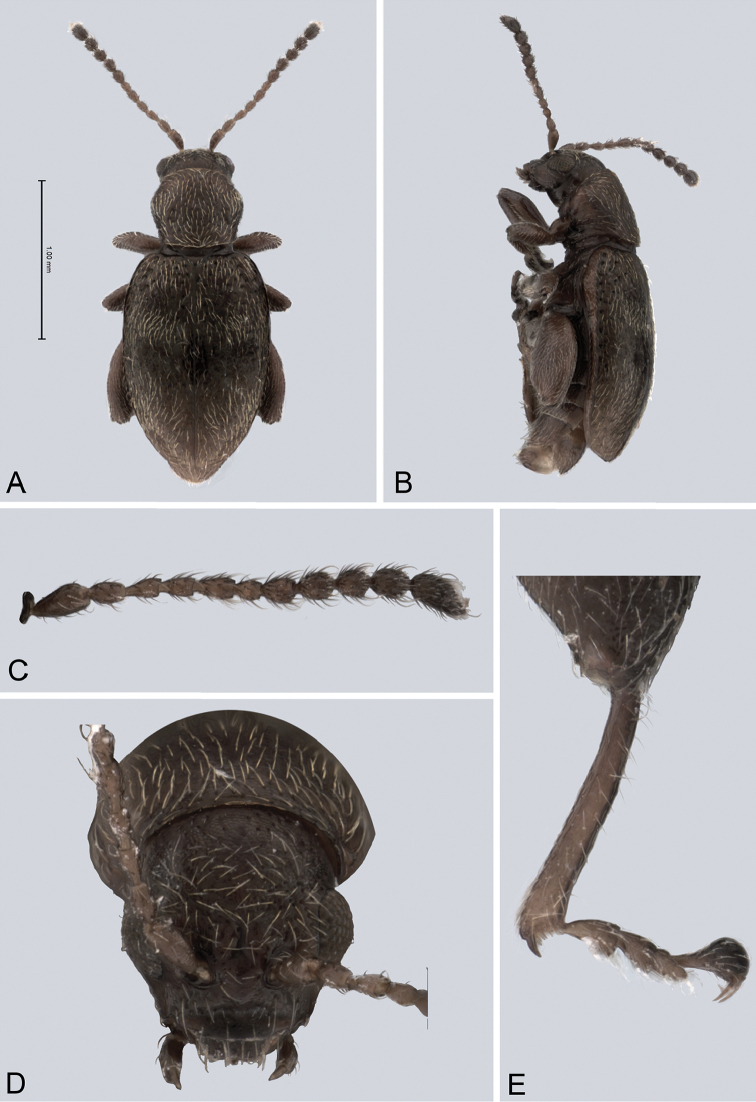
*Andersonoplatus lagunanegra*. **A** Habitus dorsal **B** Habitus lateral **C** Antenna **D** Head, frontal view **E** Hind leg.

*Head* (Fig. 15D): slightly convex in lateral view, shiny, evenly reticulated, with sparse pilosity. Frons and vertex forming nearly a 135° angle in lateral view. Vertex punctuated. Antennal callus delimited from vertex by slightly inclined sulcus; slightly elevated above vertex; surface uneven, with more than two punctures, some of them bearing setae. Orbital sulcus shallow. Supraorbital sulcus absent. Suprafrontal sulcus shallow. Frontolateral sulcus absent. Orbit narrow, punctured, as narrow as transverse diameter of antennal socket. Interantennal space wider than transverse diameter of eye and transverse diameter of antennal socket separately. Frontal ridge short and wide. Anterofrontal ridge short, relatively tall, oblique. Eyes oval. Antenna with antennomeres II-X similar in length, eleventh slightly longer, the last five moniliform; sixth antennomere much smaller than seventh.

*Thorax*: pronotum (Fig. 15A) narrower than elytra. Anterior margin wider than posterior, posterior margin slightly convex, lateral margin slightly sinuated. Surface reticulated, pilose. Pronotum with shallow, elongated impression anteromedially (absent in female). Pronotal disc not raised. Scutellum rounded, wider than long. Prosternal surface reticulated. Prosternal intercoxal process thin. Posterior end twice as wide as middle. Procoxae very close to each other. Elytra fused. Elytral surface shiny, pilose, with golden, semi-erect hairs, punctate (Fig. 15A); two inclined strips of less dense pilosity. Lines of punctures not well defined, partly confused. Shallow impression running on base of fifth and sixth striae. Epipleura nearly vertical. Metafemur enlarged, 1.38 times longer than metatibia. Metatibia almost straight in lateral and dorsal view. Outer and inner lateral dorsal ridge ending in an apical tooth followed by numerous denticles (Fig. 15E). Claws simple and long.

**Figure 16.**
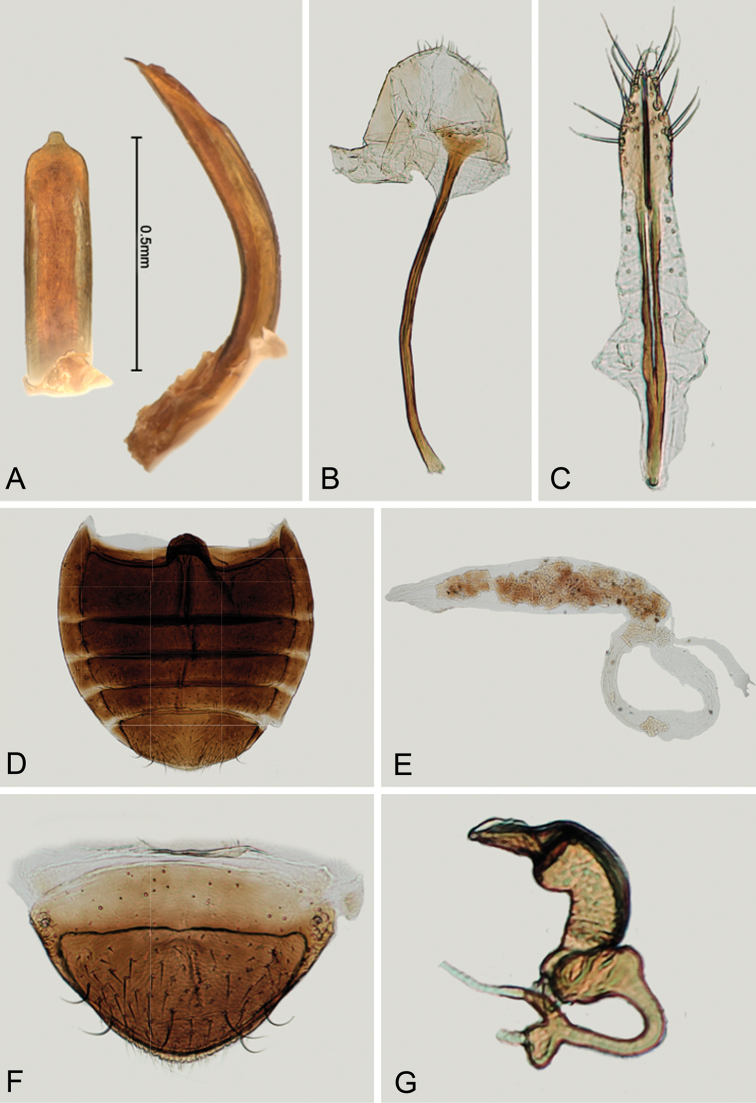
*Andersonoplatus lagunanegra*. **A** Median lobe of aedeagus, ventral and lateral views **B** Tignum **C** Vaginal palpi **D** Female abdomen, ventral view **E** Gut **F** Last abdominal tergite of female **G** Spermatheca.

*Male genitalia* (Fig. 16A): ventral side with shallow longitudinal impression running deeper basally; in lateral view strongly curved, apical denticle (in ventral view) longer and better pronounced.

*Female genitalia* (Fig. 16B–G): tignum long, narrow, slightly bent, with central canal; anterior sclerotization narrow, posterior sclerotization poorly delineated, much wider than anterior (Fig. 16B). Vaginal palpi elongate, basally strongly sclerotized, each with approximately eight setae at apex (Fig. 16C). Palpi pointed at apex, enlarged at last third but thinned at apex, situated close together and merged anteriorly for more than half of their length. Spermatheca curved, with receptacle and pump not differentiated from each other, receptacle longer than pump. Apex of pump with spoon-like projection. Spermathecal duct short, widest at base, without coils, making loop (Fig. 16G).

#### Type material.

**Holotype**, ♂. VENEZUELA: Merida/ P.N. Sierra Nevada/ Laguna Negra, 3300m/ 08°47'14"N, 70°48'31"W/ 23.V.1998-028B, R.Anderson/ elfin forest litter (MIZA). **Paratypes** (2♀ CMNC, USNM) same label as holotype except “028H”.

#### Etymology.

The specific epithet is a noun in apposition based on the type locality.

#### Differential diagnosis.

*Andersonoplatus lagunanegra* is similar to *A. saviniae* and can be separated from it based on the following characters: sixth antennomere much smaller than seventh (Fig. 15C); aedeagus in lateral view strongly curved, apical denticle (in ventral view) longer and better pronounced (Fig. 16A).

### Andersonoplatus macubaji sp. n.

Taxon classificationAnimaliaColeopteraChrysomelidae

http://zoobank.org/3696A919-D7B9-4D6A-8C3C-9FA9EB25A321

Figs 17 , 18

#### Description.

Body length 2.86–2.97 mm, width 1.40–1.51 mm, shiny, pilose, flat in lateral view. Color brown.

**Figure 17.**
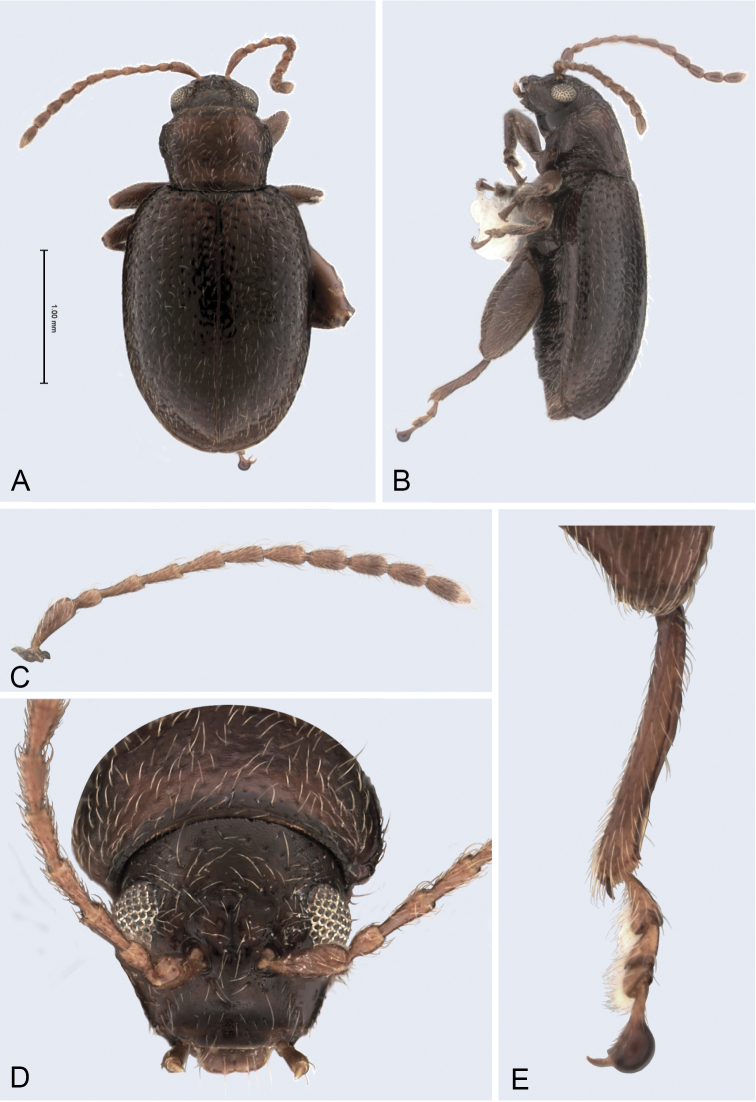
*Andersonoplatus macubaji*. **A** Habitus dorsal **B** Habitus lateral **C** Antenna **D** Head, frontal view **E** Hind leg.

*Head* (Fig. 17D): slightly convex in lateral view, shiny, evenly reticulated, vertex punctuated. Frons and vertex forming nearly a 135° angle in lateral view. Antennal callus delimited from vertex by shallow, slightly inclined supracallinal sulcus. Antennal callus slightly raised, covered with punctures bearing setae. Orbital sulcus shallow. Supraorbital sulcus absent. Supracallinal sulcus poorly delimited. Suprafrontal and frontolateral sulcus shallow. Frontogenal and frontolateral sutures well developed. Orbit as wide as transverse diameter of antennal socket. Interantennal space narrower than transverse diameter of eye and wider than transverse diameter of antennal socket. Frontal ridge short, narrow. Anterofrontal ridge short, relatively tall, oblique. Antennae filiform; second antennomere shorter.

*Thorax*: pronotum (Fig. 17A, B) narrower than elytra. Anterior margin wider than posterior, posterior margin straight, lateral margin slightly sinuated. Surface reticulate, punctate, pilose. Pronotal disc not raised. Scutellum rounded, reticulated, wider than long. Prosternal surface reticulated. Prosternal intercoxal process narrow. Posterior end twice as wide as middle. Elytra fused. Elytral surface shiny, pilose, punctate. Punctures forming nine striae. Interspaces flat. Second and third striae reaching elytral base. Epipleura nearly vertical, pilose. Metafemur 1.74 times longer than metatibia. Metatibia almost straight in lateral view, slightly curved in dorsal view. Outer and inner lateral dorsal ridge ending in an apical tooth followed by numerous denticles (Fig. 17E). Metatarsomeres one and two of similar size, twice as long than third. Claws simple and long.

Male unknown.

**Figure 18.**
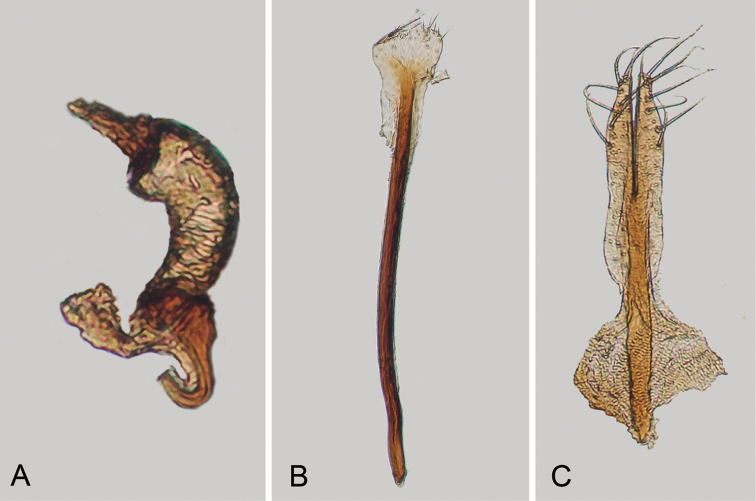
*Andersonoplatus macubaji*. **A** Spermatheca **B** Tignum **C** Vaginal palpi.

*Female genitalia* (Fig. 18A–C): tignum long, narrow, slightly bent, with central canal; anterior sclerotization narrow, posterior sclerotization poorly delineated, two-pronged pitchfork-like, wider than anterior (Fig. 18B). Vaginal palpi elongate, basally strongly sclerotized, posterior sclerotization concave. Palpi narrowly rounded at apex, enlarged at last third but thinned at apex, separated on one third of their length (Fig. 18C). Spermatheca curved, with receptacle and pump not differentiated from each other, receptacle longer than pump. Apex of pump with spoon-like projection relatively thick at base. Spermathecal duct short, widest at base, without coils, making narrow loop (Fig. 18A).

#### Type material.

**Holotype**, ♀. VENEZUELA: Merida/ Apartaderos, Laguna/ Macubaji, 3500m/ 29.VII.1989, S.&J. Peck/ paramo cushion plant/ litter, 89-285 (MIZA). **Paratype** (1♀ USNM). Same label as holotype.

#### Etymology.

The specific epithet is a noun in apposition based on the type locality.

#### Differential diagnosis.

*Andersonoplatus macubaji* is similar to *A. merida* and can be differentiated from it based on the following characters: vaginal palpi separated on one third of their length (Fig. 18C); posterior sclerotization of vaginal palpi concave on side (Fig. 18C); anterior end of tignum narrow (Fig. 18B).

### Andersonoplatus merga sp. n.

Taxon classificationAnimaliaColeopteraChrysomelidae

http://zoobank.org/AC4589C1-AA7A-4325-AF98-7147B95B053B

Figs 19 , 20

#### Description.

Body length 3.51–3.67 mm, width 1.78–1.89 mm, shiny, with sparse, semi-erect hairs, slightly convex in lateral view. Color dark brown.

**Figure 19.**
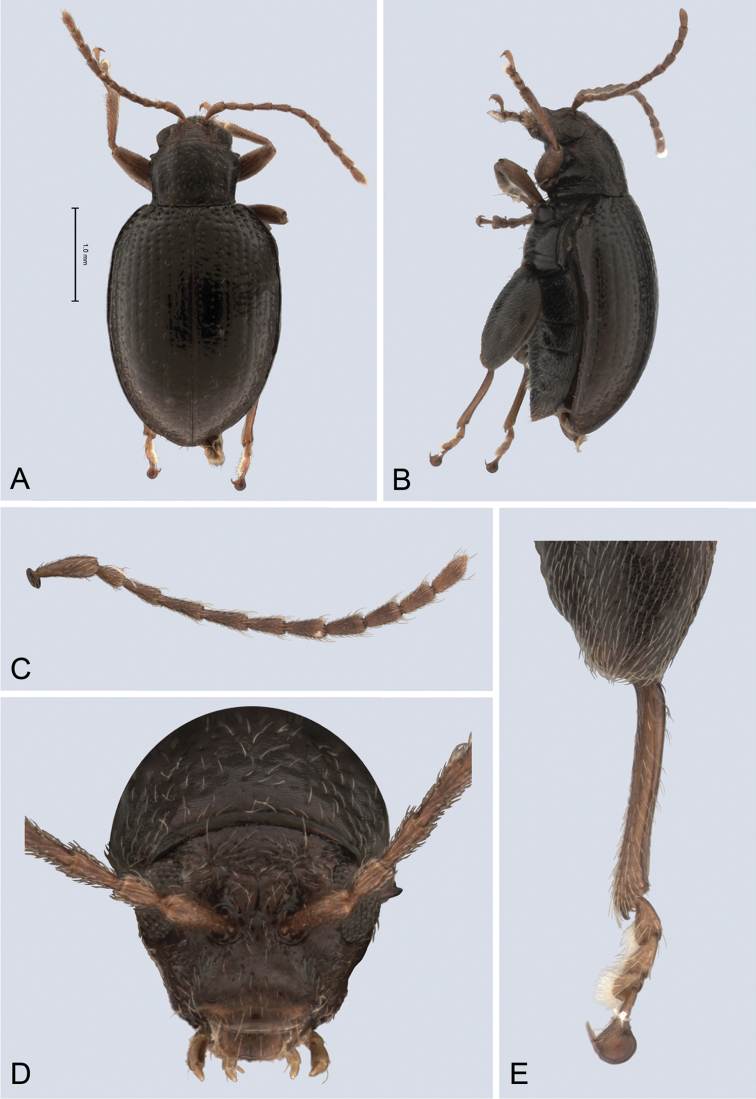
*Andersonoplatus merga*. **A** Habitus dorsal **B** Habitus lateral **C** Antenna **D** Head, frontal view **E** Hind leg.

*Head* (Fig. 19D): slightly convex in lateral view, shiny, evenly reticulated, generally sparsely punctuated. Frons and vertex forming nearly a 135° angle in lateral view. Antennal callus delimited from vertex by poorly delimited supracallinal sulcus. Antennal callus slightly raised, covered with punctures. Vertex with coarse transverse wrinkles most evident near orbital sulci. Orbital sulcus shallow. Supraorbital absent. Suprafrontal sulcus shallow. Frontolateral sulcus deep. Frontogenal suture well developed. Orbit as wide as transverse diameter of antennal socket. Interantennal space narrower than transverse diameter of eye and wider than transverse diameter of antennal socket. Frontal ridge short, V-shaped. Antenna filiform; second antennomere shorter.

*Thorax*: pronotum (Fig. 19A, B) narrower than elytra. Anterior margin wider than posterior, posterior margin almost straight, lateral margin sinuated. Anterolateral callosities long, pointed, denticle-like. Surface reticulated, punctuated, pilose. Pronotal disc slightly raised. Scutellum triangular, reticulated, wider than long. Prosternal surface reticulated. Posterior end approximately twice as wide as middle. Procoxae globose. Elytra fused. Elytral surface shiny, with very sparse and short hairs, punctate. Punctures forming nine striae. Interspaces slightly convex. Second and third striae reaching elytral base. Epipleura nearly vertical, pilose. Metafemur 1.6 times longer than metatibia. Metatibia almost straight in lateral and slightly curved in dorsal view. Metatarsomeres one and two of similar size, twice as long as third. Claws appendiculate and long.

Male unknown.

**Figure 20.**
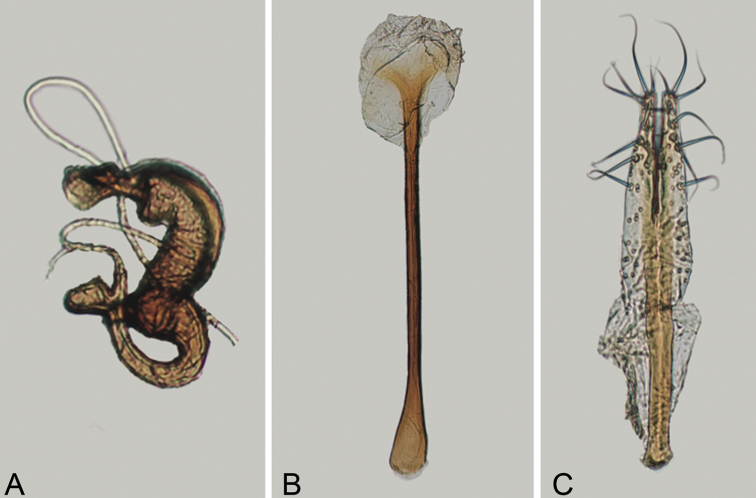
*Andersonoplatus merga*. **A** Spermatheca **B** Tignum **C** Vaginal palpi.

*Female genitalia* (Fig. 20A–C): tignum long, narrow, slightly bent, with central canal; anterior sclerotization narrow, posterior sclerotization well delineated, two-pronged pitchfork-like, wider than anterior (Fig. 20B). Vaginal palpi elongate, basally strongly sclerotized, each with approximately eight setae at apex. Palpi narrowly rounded at apex, enlarged at last third but thinned at apex; separated on one third of their length; posterior sclerotization of vaginal palpi concave on side (Fig. 20C). Spermatheca curved, with receptacle and pump not differentiated from each other, receptacle longer than pump. Apex of pump with spoon-like projection relatively thick at base. Spermathecal duct short, widest at base, without coils, making narrow loop (Fig. 20A).

#### Type material.

**Holotype**, ♀. VENEZUELA: Merida/ Paramo de La Culata/ 18.5km N.E. Merida, 2950m/ 08°44'34"N, 71°03'44"W/ 25.V.1998-037A, R.Anderson/ paramo, streamside shrub litter (MIZA). **Paratype** (1♀ USNM). Same label as holotype.

#### Etymology.

The specific epithet is a noun in apposition based on a two-pronged pitchfork-like posterior margin of tignum.

#### Differential diagnosis.

*Andersonoplatus merga* is similar to *A. macubaji* and *A. merida*. It can be separated from them based on the following characters: vertex with coarse transverse wrinkles most evident near orbital sulci (Fig. 19D); anterolateral callosity of pronotum long curved denticle-like (Fig. 19A). In *A. macubaji* and *A. merida*: vertex without coarse transverse wrinkles near orbital sulci (Fig. 17B), anterolateral callosity of pronotum short, not denticle-like (Fig. 17A).

### Andersonoplatus merida sp. n.

Taxon classificationAnimaliaColeopteraChrysomelidae

http://zoobank.org/EBFBEE6A-CB79-4E91-A8E5-849C36407F80

Figs 21 , 22

#### Description.

Body length 3.18–3.56 mm, width 1.56–1.89 mm, shiny, pilose, nearly flat in lateral view. Color castaneous.

**Figure 21.**
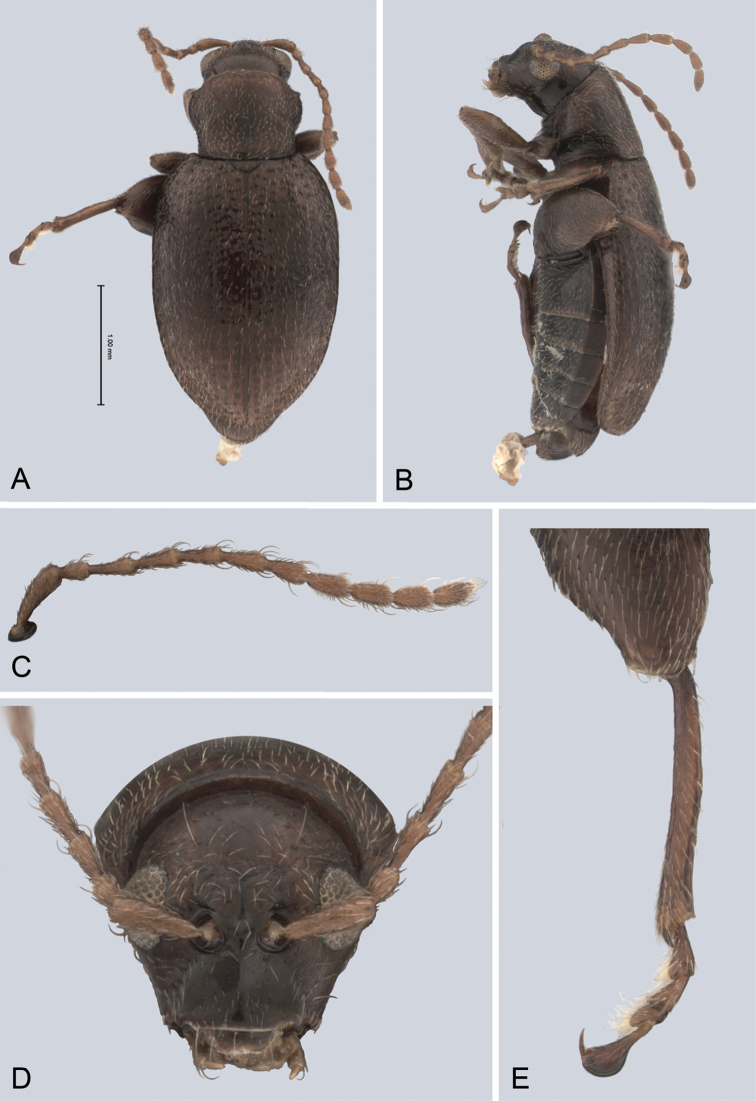
*Andersonoplatus merida*. **A** Habitus dorsal **B** Habitus lateral **C** Antenna **D** Head, frontal view **E** Hind leg.

*Head* (Fig. 21D): slightly convex in lateral view, shiny, evenly reticulated, generally sparsely punctuated. Frons and vertex forming nearly a 135° angle in lateral view. Antennal callus delimited from vertex by poorly formed, inclined supracallinal sulcus. Antennal callus slightly raised, surface uneven, with more than two punctures, some of them bearing setae. Orbital and supraorbital sulcus absent. Suprafrontal shallow. Frontolateral sulcus absent. Frontogenal suture well developed. Orbit slightly wider than transverse diameter of antennal socket. Interantennal space narrower than transverse diameter of eye and slightly wider than transverse diameter of antennal socket. Antennal socket rounded. Frontal ridge short, V-shaped. Antennae filiform; second antennomere shorter.

*Thorax*: pronotum (Figs 21A, B) narrower than elytra. Anterior margin wider than posterior, posterior margin nearly straight, lateral margin sinuated. Anterior angles pointed outwards. Surface reticulated, punctuate, pilose. Pronotal disc not raised. Scutellum triangular, reticulated, wider than long. Prosternal surface reticulated. Prosternal intercoxal process thin. Posterior end twice as wide as middle. Procoxae very close to each other. Elytra fused. Elytral surface shiny, pilose, punctate (Fig. 21A). Punctures forming nine striae. Interspaces flat. Second and third striae reaching elytral base. Epipleura nearly horizontal. Metafemur 1.37 times longer than metatibia. Metatibia almost straight in lateral and dorsal views. Metatarsomeres one and two of similar size, twice as long as third. Claws appendiculate and long.

**Figure 22.**
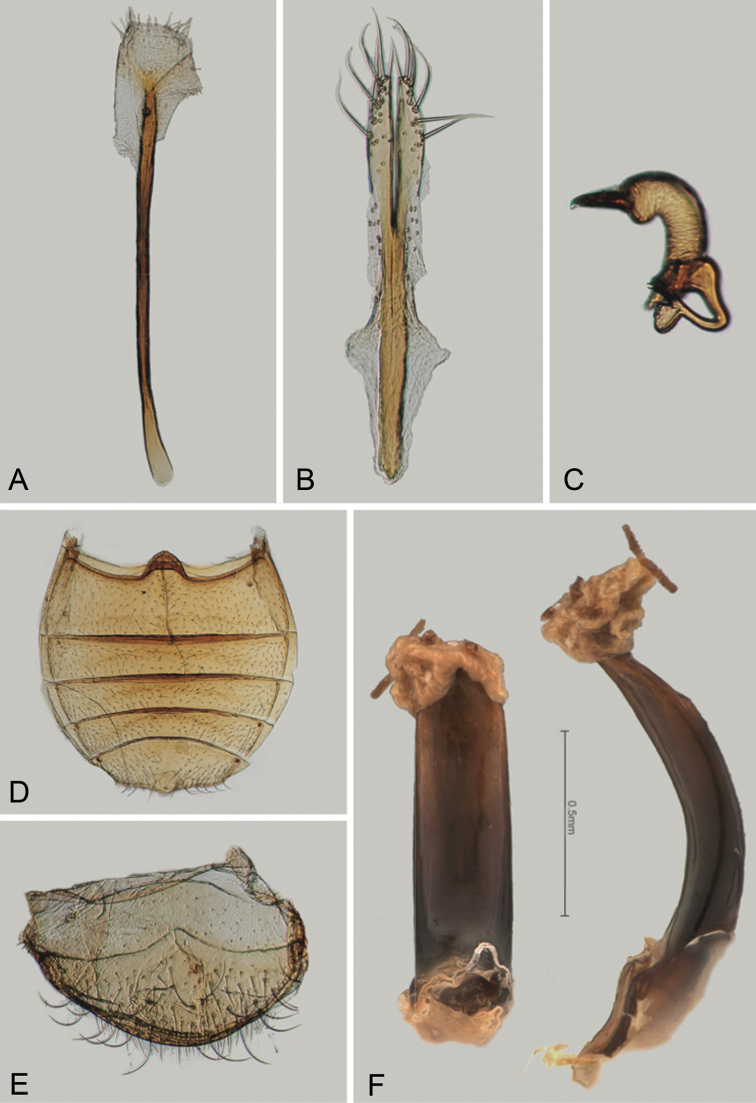
*Andersonoplatus merida*. **A** Tignum **B** Vaginal palpi **C** Spermatheca **D** Female abdomen, ventral view **E** Last abdominal tergite of female **F** Median lobe of aedeagus, ventral and lateral views.

*Male genitalia* (Fig. 22F): ventral side evenly convex without grooves and ridges, flattened apically.

*Female genitalia* (Fig. 22A–E): tignum long, narrow, slightly bent, with central canal; anterior sclerotization relatively wide, posterior sclerotization well delineated, narrower than anterior (Fig. 22A). Vaginal palpi elongate, basally strongly sclerotized, each with eight setae at apex. Palpi narrowly rounded at apex, posterior sclerotization slightly curved on side (Fig. 22B), separated on more than one third of their length (Fig. 22B). Spermatheca curved, with receptacle and pump not differentiated from each other, receptacle longer than pump. Apex of pump with relatively thick spoon-like projection. Spermathecal duct short, widest at base, without coils, making narrow loop (Fig. 22C).

#### Type material.

**Holotype**, ♂. (1) VENEZUELA: Merida/ Alto de Timotes, Paramo/ de Mucuchies, 4000m/ 08°51'30"N, 70°49'29"W/ 26.V.1998-043, R.Anderson. (2) dead leaves under /*Espeletia* sp (MIZA). **Paratypes** (2♀ USNM). (1♀ USNM) VENEZUELA: Merida/ P.N.Sierra Nevada/ Laguna Negra, 3300m/ 08°47'14"N, 70°48'31"W/ 23.V.1998-028B, R.Anderson/ elfin forest litter. (1♀ CMNC) VENEZUELA: Merida/ Merida, Telef./ Loma Redonda, 4100m/ 22–29.VI.1989, S.&J. Peck/ paramo, *Polylepsis* grove/ ex: carrion trap.

#### Etymology.

The specific epithet is a noun in apposition based on the type locality.

#### Differential diagnosis.

*Andersonoplatus merida* is similar to *A. macubaji* and can be differentiated from it based on the following characters: vaginal palpi separated on more than one third of their length (Fig. 22B); posterior sclerotization of vaginal palpi slightly curved on side (Fig. 22B); anterior end of tignum relatively wide (Fig. 22A).

### Andersonoplatus microoculus sp. n.

Taxon classificationAnimaliaColeopteraChrysomelidae

http://zoobank.org/146846F8-FD3E-41AD-A8C2-9CF7FC6A9CD0

**Figure 23.**
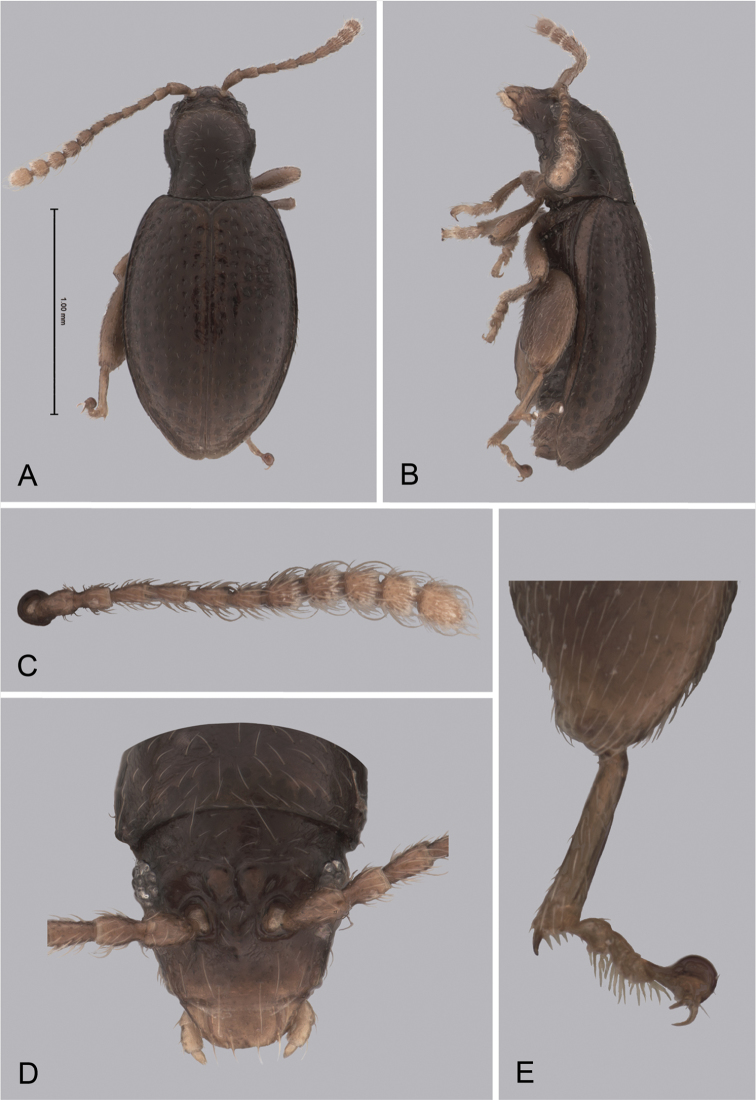
*Andersonoplatus microoculus*. **A** Habitus dorsal **B** Habitus lateral **C** Antenna **D** Head, frontal view **E** Hind leg.

**Figure 24.**
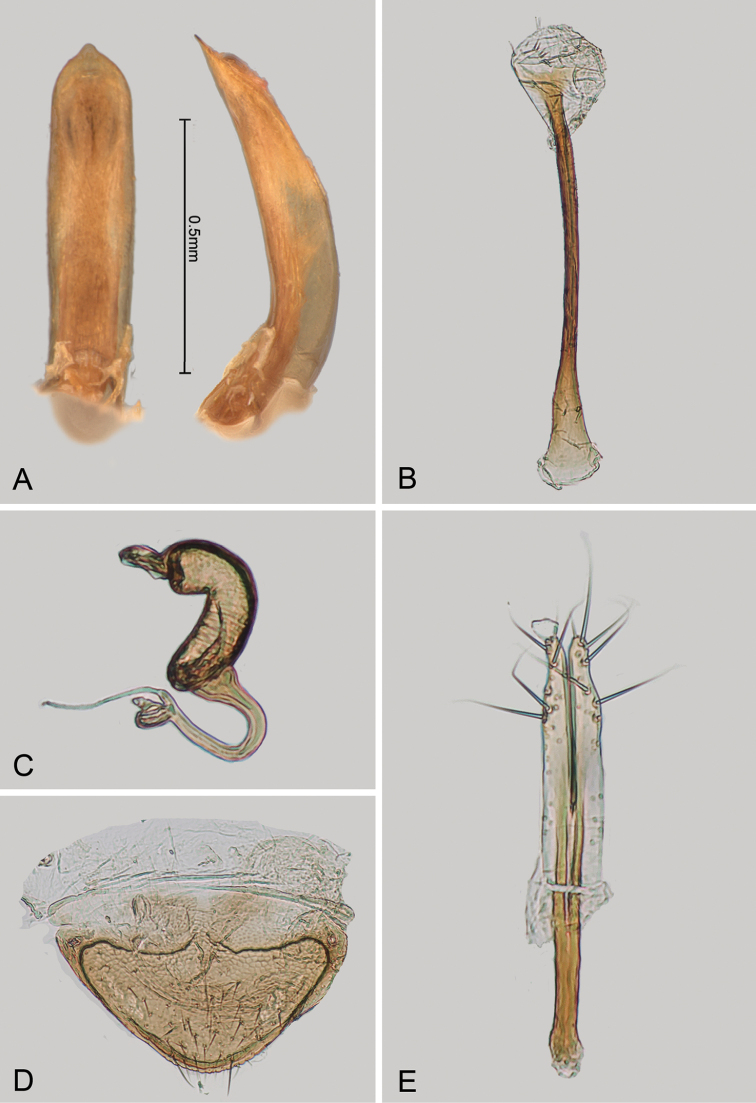
*Andersonoplatus microoculus*. **A** Median lobe of aedeagus, ventral and lateral views **B** Tignum **C** Spermatheca **D** Last abdominal tergite of female **E** Vaginal palpi.

**Figure 25.**
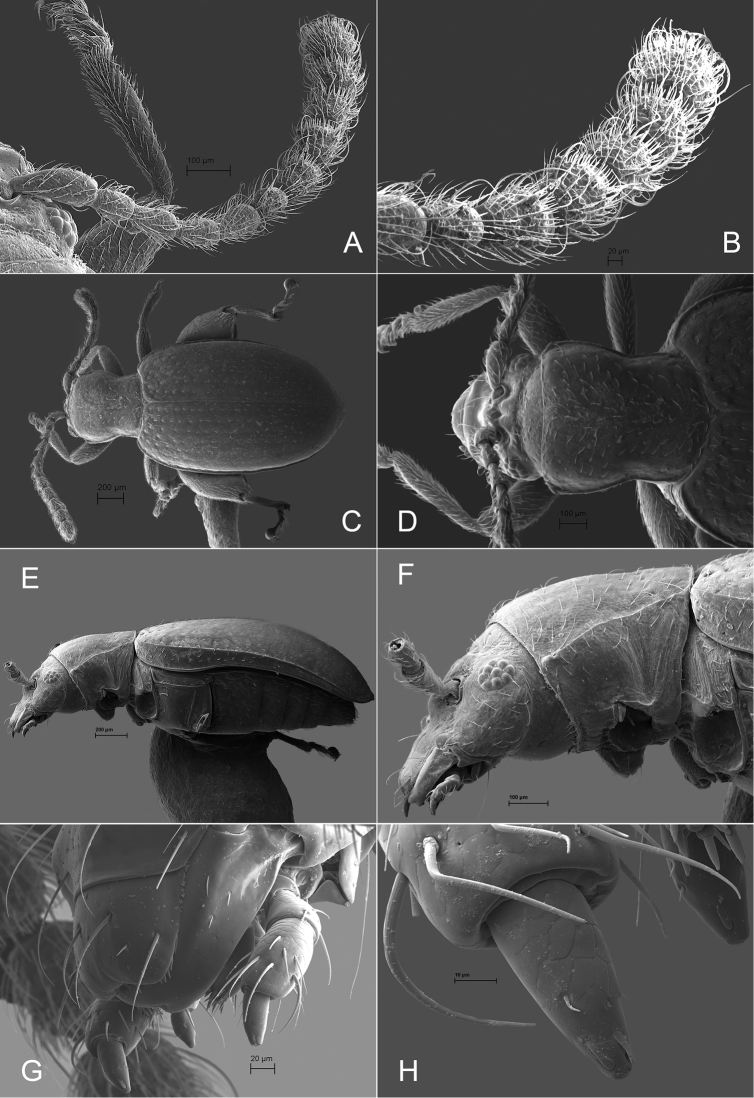
*Andersonoplatus microoculus*. **A** Antenna **B** Seven apical antennomeres **C** Habitus dorsal **D** Pronotum in dorsal view **E** Habitus lateral **F** Head and pronotum in lateral view **G** Mouth parts in lateral view **H** Last maxillary palpomere.

**Figure 26.**
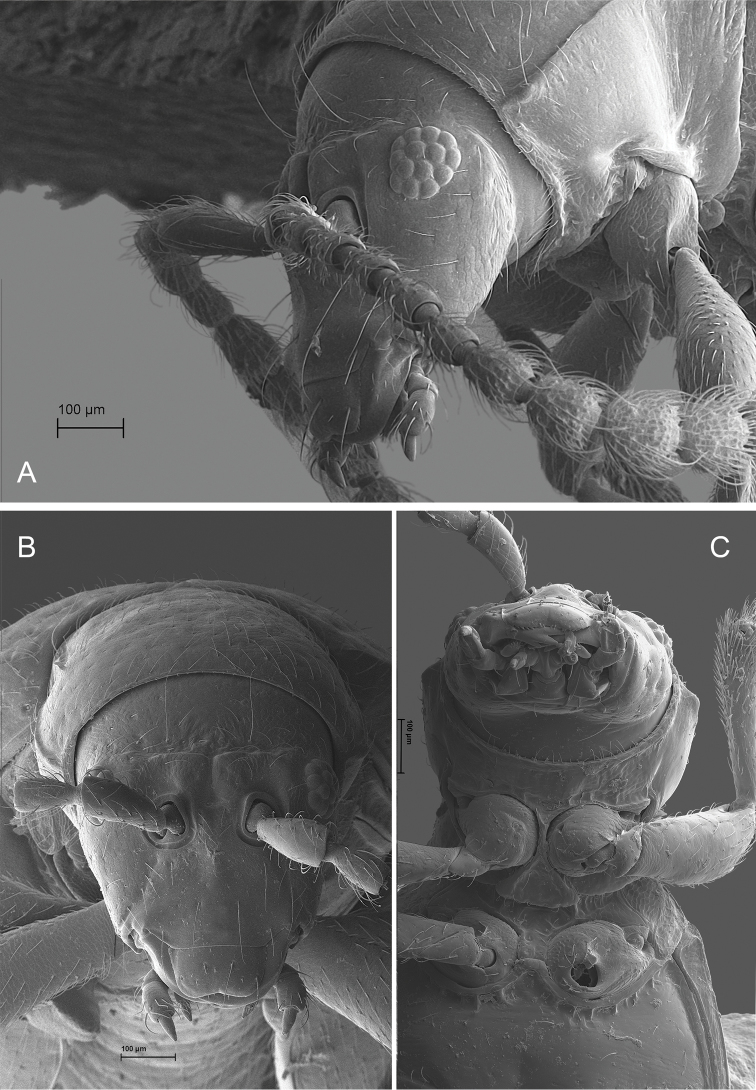
*Andersonoplatus microoculus*. **A** Head in lateral view **B** Head in frontal view **C** Head and pronotum in ventral view.

**Figure 27.**
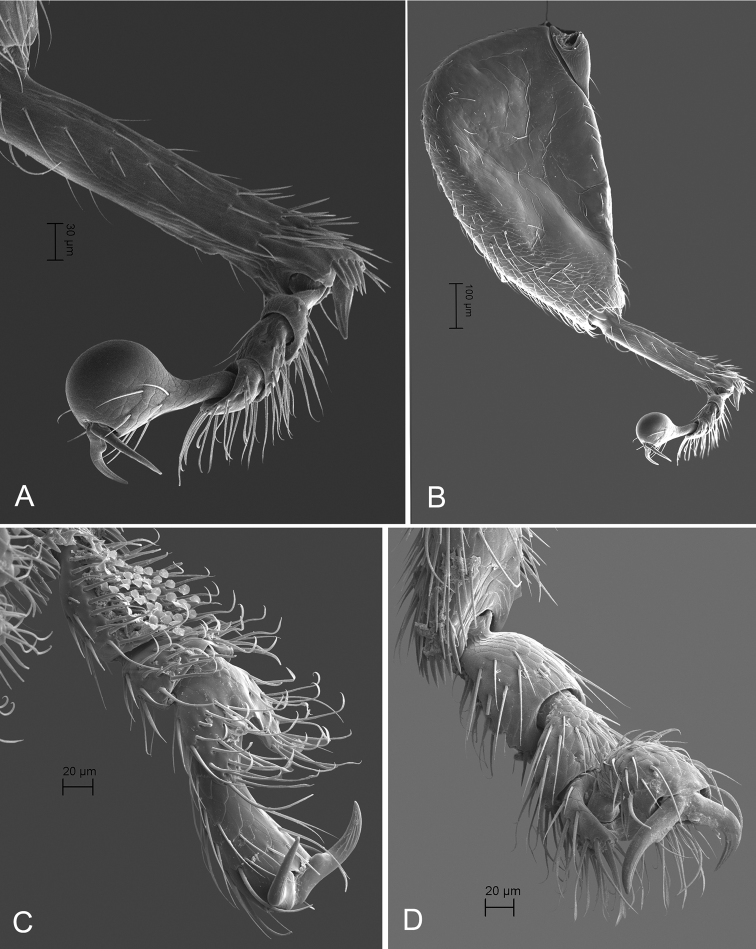
*Andersonoplatus microoculus*. **A** Hind tibia and tarsus in dorsal view **B** Hind leg in dorsal view **C** Protarsomeres in ventral view **D** Protarsomeres in dorsal view.

Figs 23 , 24 , 25 , 26 , 27

#### Description.

Body length 2.10–2.43 mm, width 0.97–1.18 mm, pronotum and elytra with sparse, semi-erect hairs, shiny, slightly flat in lateral view. Color light brown to almost black; antennae and legs yellow or at least lighter than rest of body.

*Head* (Figs 23D; 25F; 26A, B, C): flat in lateral view, generally smooth, vertex slightly reticulated; gena shiny, with very sparse pilosity. Antennal callus delimited from vertex by well-developed and straight supracallinal sulcus. Antennal callus elevated above vertex, surface even, with no or two punctures, if bearing setae, they are short. Orbital sulcus shallow. Supraorbital sulcus shallow almost connected with supracallinal sulcus. Suprafrontal sulcus shallow. Frontolateral sulcus absent. Frontogenal suture shallow. Orbit as wide as transverse diameter of antennal socket. Interantennal space wider than transverse diameter of eye and wider than transverse diameter of antennal socket. Antennal socket rounded. Frontal ridge short, V-shaped. Anterofrontal ridge low, oblique. Eyes small, with approximately 12 large ommatidia. Antenna with the last five antennomeres moniliform, with denser and longer setae.

*Thorax*: pronotum (Fig. 25D, F) longer than wide, much narrower than elytra, notched at middle. Anterior margin nearly straight, wider than posterior; posterior margin slightly convex, lateral margin sinuated. Surface reticulated, with pilosity sparse. Post basal impression absent. Pronotal disc not raised. Scutellum very small and triangular. Prosternal surface reticulated. Prosternal intercoxal process thin in middle. Posterior end more than twice as wide as middle (Fig. 26C). Elytra fused. Elytral surface shiny, with short, white, semi-erect hairs. Punctures forming seven striae. Each punctation bears one very short setae (can be found some setae on the interestriae). Interspaces flat. Second and third striae reaching elytral base. Epipleura nearly vertical. Metafemur greatly enlarged, 1.95 times longer than metatibia. Metatarsomeres one and two similar in size, slightly longer than third. Claws slightly appendiculate and long (Fig. 27C, D).

*Male genitalia* (Fig. 24A): ventral side convex and shiny, without longitudinal impression, slightly flattened at apex; apical denticle well developed in ventral view, narrow, apex straight and not bent ventrally.

*Female genitalia* (Fig. 24B–E): tignum long, narrow, slightly bent, with central canal; anterior sclerotization widening gradually with curved sides and convex apex, posterior sclerotization poorly delineated, narrow, as wide as anterior (Fig. 24B). Vaginal palpi elongate, basally strongly sclerotized, each with eight setae at apex (Fig. 24E). Palpi pointed at apex, enlarged at last third but thinned at apex, situated close together and merged anteriorly for more than half of their length. Spermatheca curved, with receptacle and pump not differentiated from each other. Apex of pump with spoon-like projection. Spermathecal duct short, widest at base, without coils (Fig. 24C). Last abdominal sternite (Fig. 24D) evenly sclerotized with evenly placed setae.

#### Type material.

**Holotype**, ♂. VENEZUELA: Trujillo/ camino viejo a Trujillo, Paramo/ La Cristalina, km 9.7, 2400m/ 09°21'22"N, 70°17'51"W/ 20.V.1998-022B/ R.Anderson, elfin for. Litter (MIZA). **Paratypes** (16♂ 7♀). (5♂1♀) same label as holotype except: (2♂4♀ USNM) “022D”; (3♂1♀ USNM) “022E”; (2♂1♀ CMNC) “022F”; (3♂ USNM) “022G”; (1♂ USNM) “022J”.

#### Etymology.

The specific epithet is a noun in apposition based on relatively small eyes of the beetles.

#### Differential diagnosis.

*Andersonoplatus microoculus* can be identified by the small eyes, with approximately 12 large ommatidia (Figs 23B, D) and pronotum comparatively narrow (Fig. 23A).

### Andersonoplatus peck sp. n.

Taxon classificationAnimaliaColeopteraChrysomelidae

http://zoobank.org/B35E4FF6-541D-4124-8EFD-F533B99B32D1

Figs 28 , 29

#### Description.

Body length 1.62–1.78 mm, width 0.81–0.91 mm, shiny, pilose, with semi-erect hairs, flat in lateral view. Color light brown.

**Figure 28.**
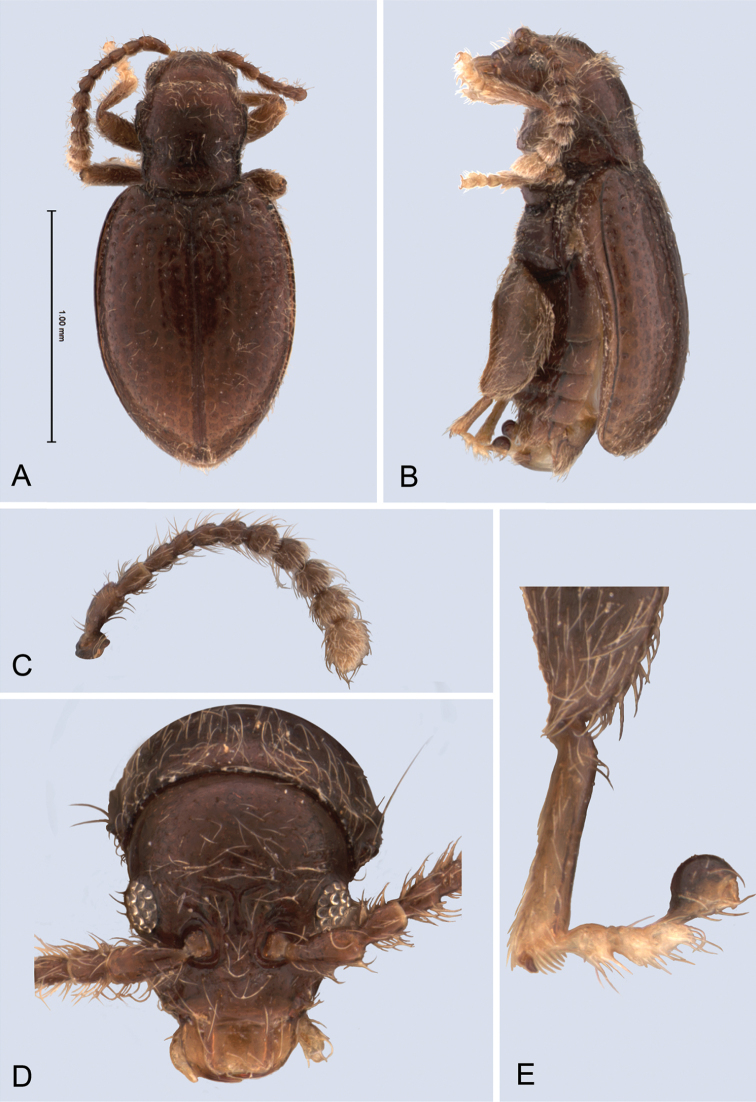
*Andersonoplatus peck*. **A** Habitus dorsal **B** Habitus lateral **C** Antenna **D** Head, frontal view **E** Hind leg.

*Head* (Fig. 28D): slightly convex in lateral view, shiny, generally reticulated, pilose. Frons and vertex at same level in lateral view. Supraorbital pore indistinguishable. Antennal callus delimited from vertex by deep and curved sulcus; not raised; surface uneven, with more than two punctures, some of them bearing setae. Orbital sulcus shallow. Supraorbital absent. Suprafrontal sulcus shallow. Frontolateral sulcus absent. Frontogenal suture well developed. Orbit narrow, nearly two times narrower than transverse diameter of antennal socket. Interantennal space slightly narrower than transverse diameter of eye and as wide as transverse diameter of antennal socket. Frontal ridge wider between antennal sockets abruptly narrowing ventrally. Anterofrontal ridge long, relatively tall, oblique. Eyes very small. Antenna with antennomeres III-XI shorter than second; last five antennomeres wider than preceding ones, moniliform (Fig. 28C).

*Thorax*: pronotum (Fig. 35A, B) longer than wide, narrower than elytra, notched at middle. Anterior margin wider than posterior, posterior margin slightly convex, lateral margin sinuated. Surface reticulated, pilose with disordered hair. Post basal impression deep, with deep rounded impressions laterally, along notch. Pronotal disc slightly raised. Scutellum rounded, much shorter than wide. Prosternal surface reticulated. Prosternal intercoxal narrow. Posterior end twice as wide as middle. Elytra fused. Elytral surface shiny, pilose, with, semi-erect, disordered hairs, punctate (Fig. 35A). Punctures forming seven striae. Interspaces slightly convex. Second stria reaching elytral base, third stria missing few punctures before elytral base. Epipleura nearly horizontal. Metafemur very enlarged, 2.01 times longer than metatibia. Metatibia almost straight in lateral view, slightly curved in dorsal view. Claws simple and long (Fig. 28E).

**Figure 29.**
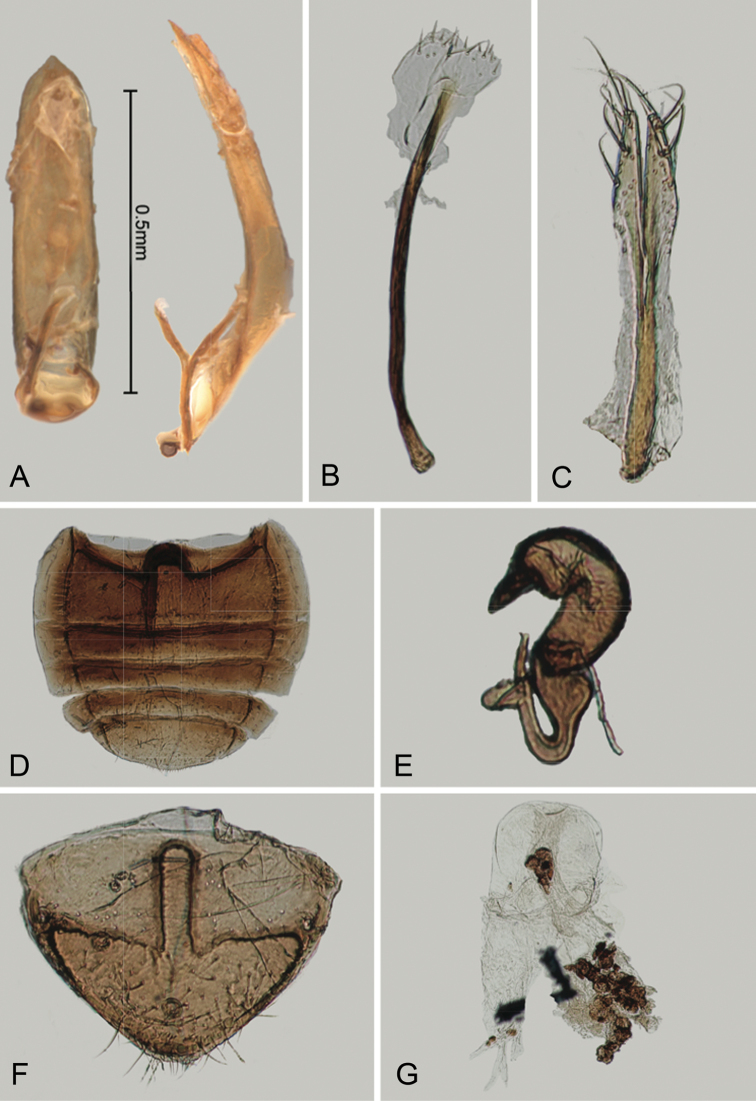
*Andersonoplatus peck*. **A** Median lobe of aedeagus, ventral and lateral views **B** Tignum **C** Vaginal palpi **D** Female abdomen, ventral view **E** Spermatheca **F** Last abdominal tergite of female **G** Gut.

*Male genitalia* (Fig. 29A): ventral side with shallow longitudinal impression running deeper basally; apical denticle well developed, long, apex straight except very tip that faces ventrally.

*Female genitalia* (Fig. 29B–G): tignum long, narrow, slightly bent, with central canal; anterior sclerotization narrow, posterior sclerotization well delineated, narrower than anterior (Fig. 29B). Vaginal palpi elongate, basally strongly sclerotized, each with approximately eight setae at apex (Fig. 29C). Palpi pointed at apex, enlarged at last third but thinned at apex, situated close together and merged anteriorly for more than half of their length. Posterior sclerotization of vaginal palpi with straight sides. Spermatheca curved, with receptacle and pump not differentiated from each other, receptacle longer than pump. Apex of pump with relatively thick spoon-like projection. Spermathecal duct short, widest at base, without coils, making narrow loop (Fig. 29E).

#### Type material.

**Holotype**, ♂. VENEZUELA: Merida/ ULA. Biol.Res. LaCarbonerra/ 20km SE Azulita, 28.VI.1989/ 2300m, S.&J. Peck/ Podocarp./ for. litter. 89-240 (MIZA). **Paratype** (1♀ USNM). Same label as holotype.

#### Etymology.

We dedicate this species to Jarmila and Stuart Peck who collected the type series. The specific epithet is a noun in apposition.

#### Differential diagnosis.

*Andersonoplatus peck* is similar to *A. baru* and can be differentiated from it based on the following characters: body 1.62–1.78 mm in length, light brown, vertex sparsely covered with setae; posterior sclerotization of vaginal palpi with straight sides; posterior sclerotization of tignum narrower than anterior. In *A. baru* body is 3.39–3.40 mm, uniformly yellow, vertex densely covered with setae; posterior sclerotization of vaginal palpi with curved sides; posterior sclerotization of tignum wider than anterior.

### Andersonoplatus rosalesi sp. n.

Taxon classificationAnimaliaColeopteraChrysomelidae

http://zoobank.org/231080EF-53DD-487D-9C3B-0D7F4C4C4A62

Figs 30 , 31

#### Description.

Body length 2.05–2.16 mm, width 1.08–1.18 mm, shiny, pilose, with semi-erect hairs, moderately convex in lateral view. Color castaneous.

**Figure 30.**
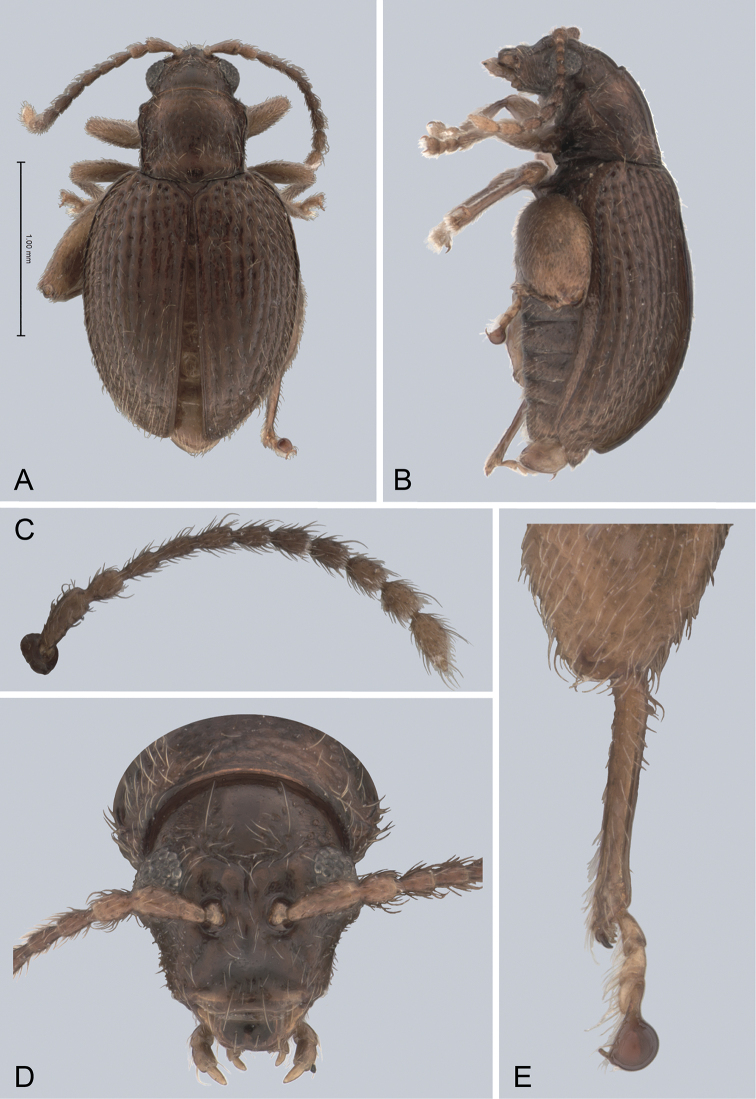
*Andersonoplatus rosalesi*. **A** Habitus dorsal **B** Habitus lateral **C** Antenna **D** Head, frontal view **E** Hind leg.

*Head* (Fig. 30D): slightly convex in lateral view, shiny, generally reticulated, with sparse pilosity. Frons and vertex forming nearly a 135° angle in lateral view. Antennal callus delimited from vertex by deep and slightly curved upward supracalinal sulcus. Antennal callus slightly raised, surface even, with no or two punctures, if bearing setae, they are short. Orbital sulcus shallow. Supraorbital sulcus absent. Suprafrontal and frontolateral sulci shallow. Frontogenal suture deep. Orbit narrow, as wide as transverse diameter of antennal socket. Interantennal space narrower than transverse diameter of eye and as wide as transverse diameter of antennal socket. Frontal ridge short and narrow. Antennae filiform; antennomeres III-XI similar in length, the last five antennomeres slightly wider than preceding ones.

*Thorax*: pronotum (Fig. 30A, B) slightly trapezoidal, almost quadrate, narrower than elytra. Anterior margin wider than posterior, posterior margin slightly concave, lateral margin almost straight. Anterior angles pointed outwards. Surface reticulated, punctuated, pilose, densely covered with well-defined punctures, diameter of which larger than distance between punctures. Pronotal disc weakly raised. Scutellum rounded, shorter than wide. Prosternal intercoxal process narrow. Posterior end nearly twice as wide as middle. Elytra not fused. Elytral surface shiny, with white, semi-erect hairs, deeply punctate (Fig. 30A, B). Punctures forming nine striae, the ninth stria is overlapping marginal one. Interspaces very convex. Second and third striae reaching elytral base. Third and fourth striae merge at apical 2/3^rd^. Epipleura slightly convex, pilose, nearly vertical, slightly narrowed at elytral apex. Metafemur greatly enlarged, 1.10 times longer than metatibia. Claws appendiculate and long.

**Figure 31.**
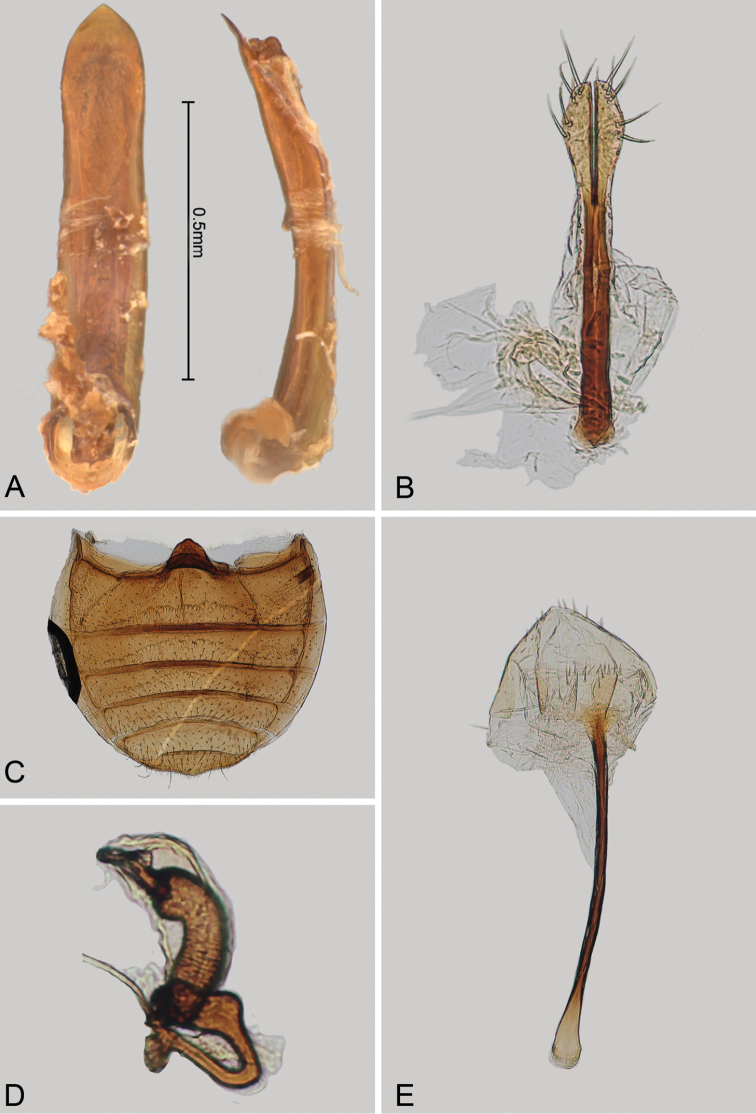
*Andersonoplatus rosalesi*. **A** Median lobe of aedeagus, ventral and lateral views **B** Vaginal palpi **C** Female abdomen, ventral view **D** Spermatheca **E** Tignum.

*Male genitalia* (Fig. 31A): ventral side flat apically, with longitudinal impression basally; apical denticle poorly developed, apex bent ventrally.

*Female genitalia* (Fig. 31B–E): tignum long, narrow, slightly bent, with central canal; anterior sclerotization widening gradually with slightly curved sides and convex apex, posterior sclerotization poorly delineated, wide, wider than anterior (Fig. 31E). Vaginal palpi elongate, basally strongly sclerotized, each with approximately eight setae at apex (Fig. 31B). Palpi pointed at apex, enlarged at last third but thinned at apex, situated close together and merged anteriorly for more than half of their length. Spermatheca curved, with receptacle and pump not differentiated from each other, receptacle longer than pump. Apex of pump with spoon-like projection. Spermathecal duct short, widest at base, without coils, making long and narrow loop (Fig. 31D).

#### Type material.

**Holotype**, ♂. VENEZUELA: Merida/ 34km N.W. Merida, Finca/ ‘Fundo La Trinidad’, 2350m/ 08°37'00"N, 71°20'12"W/ 22.V.1998-027C, R.Anderson/ montane forest litter (MIZA). **Paratypes** (5♂ USNM, CMNC, 1♀ USNM). Same labels as holotype.

#### Etymology.

We name this species after Carlos Rosales of Museo del Instituto de Zoologia, UCV, Maracay, Venezuela, a fellow coleopterist who contributed greatly to our knowledge of beetles of Venezuela.

#### Differential diagnosis.

*Andersonoplatus rosalesi* is similar to *A. andersoni*, *A. flavus* and *A. sanare* and can be separated from them based on the following characters: pronotal surface densely covered with well-defined punctures, diameter of which larger than distance between punctures (Fig. 30A) and second elytral stria reaching base of elytron (Fig. 30A).

### Andersonoplatus sanare sp. n.

Taxon classificationAnimaliaColeopteraChrysomelidae

http://zoobank.org/6D9402CE-5913-4160-B0CF-70F94EBEF0AC

Figs 32 , 33

#### Description.

Body length 2.43–3.24 mm, width 1.24–1.59 mm, shiny, pilose, with semi-erect hairs, moderately convex in lateral view. Color light brown to dark brown.

**Figure 32.**
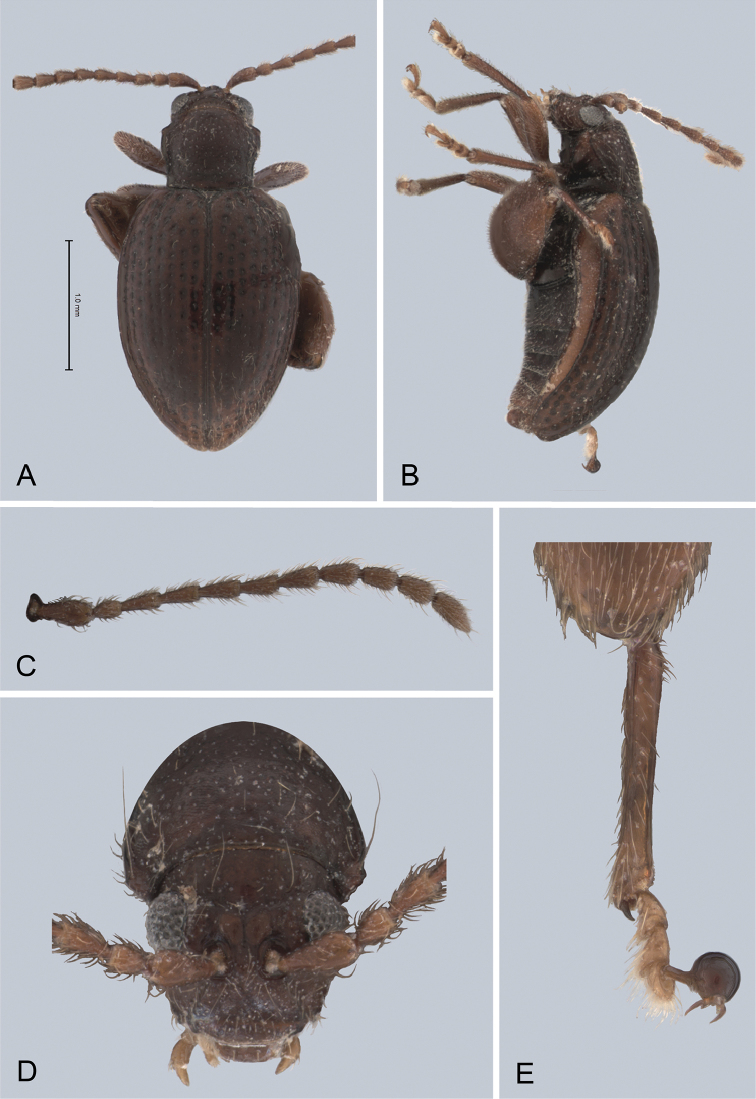
*Andersonoplatus sanare*. **A** Habitus dorsal **B** Habitus lateral **C** Antenna **D** Head, frontal view **E** Hind leg.

*Head* (Fig. 32D): slightly convex in lateral view, generally reticulated, with sparse pilosity, gena slightly punctured. Frons and vertex forming nearly a 135° angle in lateral view. Antennal callus delineate from vertex by deep and curved supracalinal sulcus. Antennal callus slightly elevated above vertex, surface even, with no or two punctures, if bearing setae, they are short. Orbital sulcus deep. Supraorbital sulcus shallow. Supraorbital and supracallinal sulci not connected. Suprafrontal sulcus shallow. Frontolateral sulcus shallow. Frontogenal suture deep. Orbit wider than transverse diameter of antennal socket. Interantennal space narrower than transverse diameter of eye and as wide as transverse diameter of antennal socket. Frontal ridge short and narrow. Antenna filiform; last five antennomeres slightly wider than preceding ones; second antennomere shorter (Fig. 32C).

*Thorax*: pronotum (Fig. 32A) much narrower than elytra. Anterior margin wider than posterior, posterior margin nearly straight, lateral margin slightly sinuated. Surface reticulated, slightly punctated, pilose, sparsely covered with variously defined punctures, diameter of which smaller than distance between punctures. Post basal impression absent in females. Pronotal disc weakly raised. Scutellum triangular, shorter than wide. Prosternal surface reticulated. Prosternal intercoxal process thin. Posterior end twice as wide as middle. Procoxae very close to each other. Elytra fused. Elytral surface shiny, with white, semi-erect hairs, punctate (Fig. 32A, B). Punctures forming nine striae, ninth stria almost merge with marginal one. Interspaces convex. Marginal line of elytra interrupted at base. Second and third striae not reaching elytral base. Epipleura slightly convex, nearly vertical, slightly narrowed at elytral apex. Metafemur greatly enlarged, 1.66 times longer than metatibia. Claws appendiculate and long (Fig. 32E).

**Figure 33.**
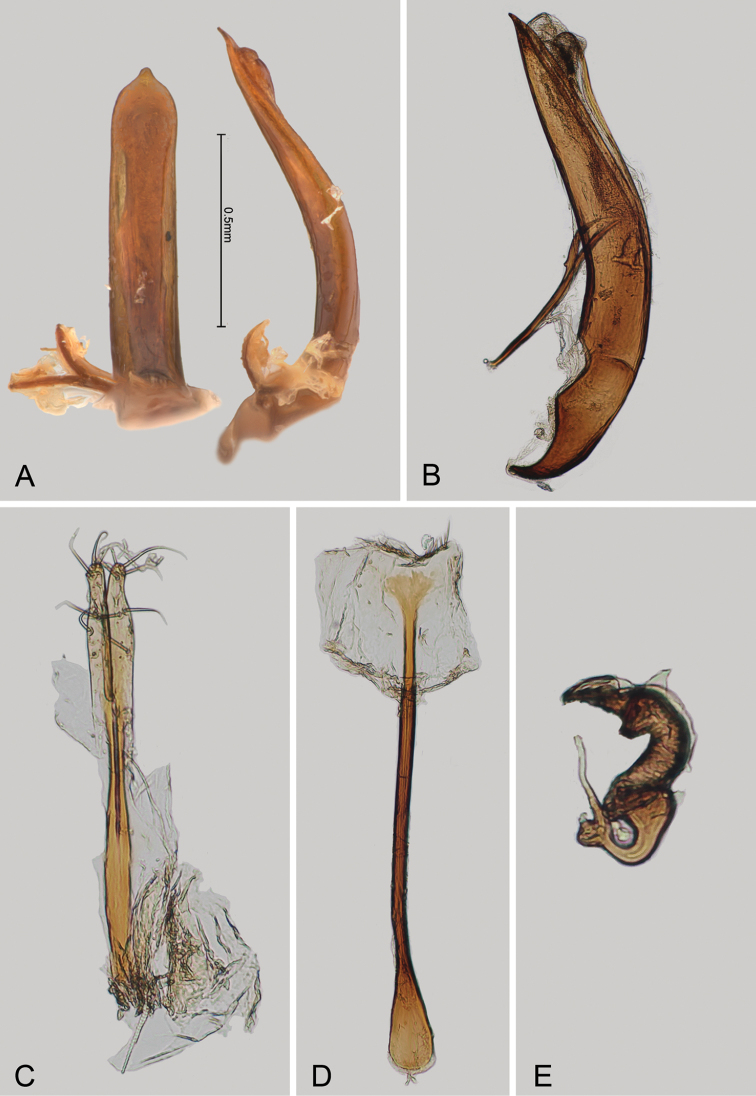
*Andersonoplatus sanare*. **A** Median lobe of aedeagus, ventral and lateral views **B** Median lobe of aedeagus, internal structures under compound microscope **C** Vaginal palpi **D** Tignum **E** Spermatheca.

*Male genitalia* (Fig. 33A, B): ventral side convex and shiny, without longitudinal impression, slightly flattened at apex; apical denticle well developed, long in ventral view, apex slightly bent ventrally.

*Female genitalia* (Fig. 33C–E): tignum long, narrow, slightly bent, with central canal; anterior sclerotization widening gradually with slightly curved sides and convex apex, posterior sclerotization poorly delineated, narrow, as wide as anterior (Fig. 33D). Vaginal palpi elongate, basally strongly sclerotized, each with approximately eight setae at apex (Fig. 33C). Palpi pointed at apex, enlarged at last third but thinned at apex, situated close together and merged anteriorly for more than half of their length. Spermatheca curved, with receptacle and pump not differentiated from each other. Apex of pump with spoon-like projection. Spermathecal duct short, widest at base, without coils, making small loop (Fig. 33E).

#### Type material.

**Holotype**, ♂. VENEZUELA: Lara/ P.N.Yacambu, 6.4km/ S.E. Sanare, 1850m/ 09°41'51"N, 69°38'57"W/ 17.V.1998-014C/ R.Anderson, cloud for. Litter (MIZA). **Paratypes** (9♂ 12♀ USNM). (5♂4♀ USNM) Same label as holotype except: (1♂1♀ CMNC) “014A”; (2♂6♀ USNM) “014E”; (1♂1♀ CMNC) “10.4km”, “1800m”, “013B”.

#### Etymology.

The specific epithet is a noun in apposition based on the type locality.

#### Differential diagnosis.

*Andersonoplatus sanare* is similar to *A. andersoni* but can be differentiated from it based on the following characters: ventral side of median lobe without longitudinal impression (Fig. 33A); spermathecal duct making relatively short loop (Fig. 33E).

### Andersonoplatus saviniae sp. n.

Taxon classificationAnimaliaColeopteraChrysomelidae

http://zoobank.org/C96450F3-D40D-4B4D-933F-831E522AB458

Figs 34 , 35

#### Description.

Body length 2.54–3.02 mm, width 1.18–1.40 mm, shiny, pilose, with semi-erect hairs, flat in lateral view. Color light brown with elytra darker (almost always in males or with band in middle in females).

**Figure 34.**
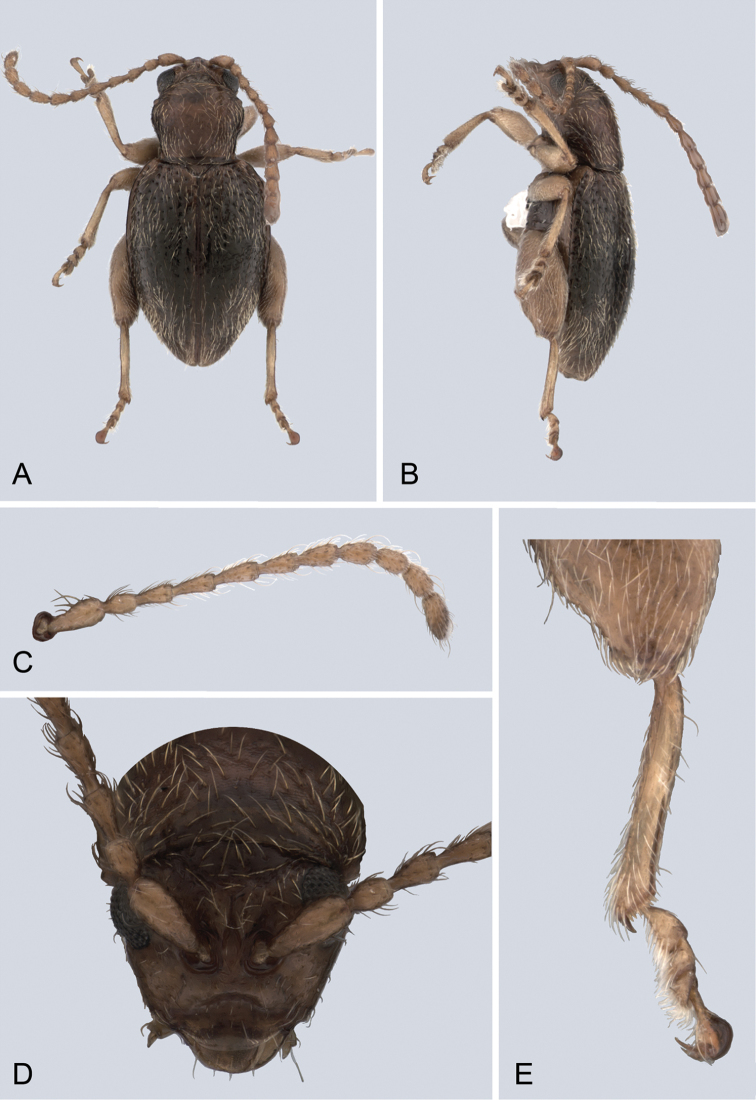
*Andersonoplatus saviniae*. **A** Habitus dorsal **B** Habitus lateral **C** Antenna **D** Head, frontal view **E** Hind leg.

*Head* (Fig. 34D): slightly flat in lateral view, shiny, generally reticulated, with sparse pilosity. Vertex covered with large, poorly defined punctures. Frons and vertex almost at same level in lateral view. Antennal callus delimited from vertex by shallow and slightly inclined supracallinal sulcus; slightly raised above vertex; surface uneven, with more than two punctures, some of them bearing setae. Orbital and supraorbital sulci shallow, represented by punctures. Suprafrontal and frontolateral sulcus shallow. Frontogenal suture shallow. Orbit narrow, punctured, narrower than transverse diameter of antennal socket. Interantennal space narrower than transverse diameter of eye and transverse diameter of antennal socket separately. Frontal ridge short and narrow. Anterofrontal ridge short, relatively tall, oblique. Antenna filiform; the last five antennomeres slightly wider and shorter than preceding ones; second antennomere shortest; sixth antennomere as long as seventh (Fig. 34C).

*Thorax*: pronotum (Fig. 34A, B) narrower than elytra. Anterior margin wider than posterior, posterior margin slightly convex, lateral margin slightly sinuated. Anterior and posterior angles pointed outwards. Surface reticulated, punctured, pilose. Pronotal disc not raised. Scutellum triangular, wider than long. Prosternal surface reticulated. Prosternal intercoxal process thin. Posterior end twice as wide as middle. Procoxae very close to each other. Elytra weakly fused. Elytral surface shiny, pilose, with white, semi-erect hairs, punctate (Fig. 34A, B). Punctures forming nine striae, ninth stria almost merge with marginal one. Interspaces slightly convex. Distinct impression running on base of fifth and sixth striae. Second and third striae reaching elytral base. Epipleura nearly vertical. Metafemur greatly enlarged, 1.33 times longer than metatibia. Metatibia almost straight in lateral view, slightly curved in dorsal view. Claws simple and long (Fig. 34E).

**Figure 35.**
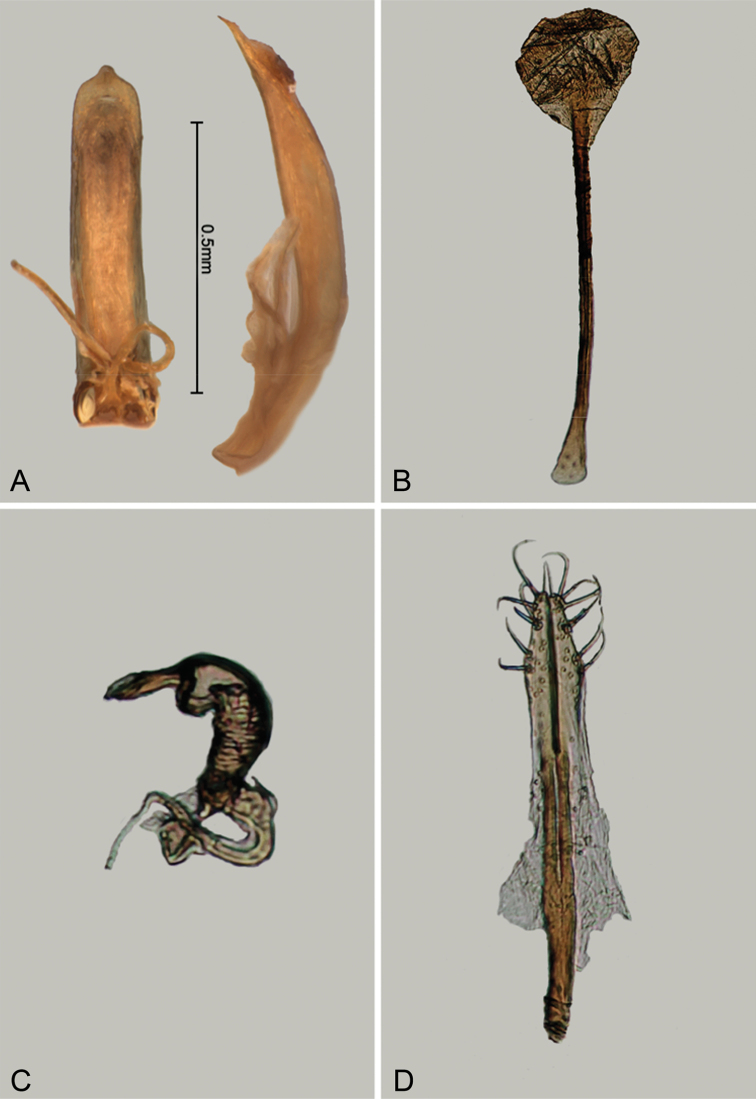
*Andersonoplatus saviniae*. **A** Median lobe of aedeagus, ventral and lateral views **B** Tignum **C** Spermatheca **D** Vaginal palpi.

*Male genitalia* (Fig. 35A): ventral side flat with shallow longitudinal impression interrupted in middle; apex bent ventrally, in lateral view nearly straight, apical denticle (in ventral view) shorter and less differentiated.

*Female genitalia* (Fig. 35B–D): tignum long, narrow, slightly bent, with central canal; anterior sclerotization widening gradually with slightly curved sides and convex apex, posterior sclerotization poorly delineated, wide, wider than anterior (Fig. 35B). Vaginal palpi elongate, basally strongly sclerotized, each with approximately eight setae at apex (Fig. 35D). Palpi pointed at apex, enlarged at last third but thinned at apex, situated close together and merged anteriorly for more than half of their length. Spermatheca curved, with receptacle and pump not differentiated from each other, receptacle longer than pump. Apex of pump with spoon-like projection. Spermathecal duct short, widest at base, without coils, making loop (Fig. 35C).

#### Type material.

**Holotype**, ♂. VENEZUELA: Trujillo/ camino viejo a Trujillo, Paramo/ La Cristalina, km 9.7, 2400m/09°21'21"N, 70°17'51"W/ 20.V.1998-022A/ R.Anderson, elfin for. Litter (MIZA). **Paratypes** (3♂ 3♀ USNM). Same label as holotype except: (1♂1♀ CMNC) “022E”; (2♂ USNM) “022F”; (2♀ USNM) “022J”.

#### Etymology.

We name this species after Vilma Savini of Museo del Instituto de Zoologia, UCV, Maracay, Venezuela, a fellow coleopterist who contributed greatly to our knowledge of beetles of Venezuela.

#### Differential diagnosis.

*Andersonoplatus saviniae* is similar to *A. lagunanegra* and can be differentiated from it based on the following characters: sixth antennomere as long as seventh (Fig. 34C); aedeagus in lateral view nearly straight, apical denticle (in ventral view) shorter and less differentiated (Fig. 35A).

##### Key to *Andersonoplatus* species

**Table:** 

1	Surface of antennal calli uneven, with more than two punctures, some of them bearing long setae	**2**
–	Surface of antennal calli even, with no or two punctures, if bearing setae, they are short	**8**
2	Elytral striae well recognized, often placed in grooves making interspaces convex	**3**
–	Elytral striae poorly recognized, punctures not in grooves making, interspaces flat	**7**
3	Supracallinal sulcus sharply delimited	**4**
–	Supracallinal sulcus poorly delimited	**5**
4	Body 1.62–1.78 mm in length, light brown, vertex sparsely covered with setae. Posterior sclerotization of vaginal palpi with straight sides. Posterior sclerotization of tignum narrower than anterior	***Andersonoplatus peck***
–	Body 3.39–3.40 mm, uniformly yellowish, vertex densely covered with setae. Posterior sclerotization of vaginal palpi with curved sides. Posterior sclerotization of tignum wider than anterior	***Andersonoplatus baru***
5	Vertex with coarse transverse wrinkles most evident near orbital sulci (Fig. 19D). Anterolateral callosity of pronotum long curved denticle-like (Fig. 19A). Body dark in color (Fig. 19A)	***Andersonoplatus merga***
–	Vertex without coarse transverse wrinkles near orbital sulci (Fig. 17B). Anterolateral callosity of pronotum short, not denticle-like (Fig. 17A). Body lighter in color (Fig. 17A)	**6**
6	Vaginal palpi separated on one third of their length (Fig. 18C). Posterior sclerotization of vaginal palpi concave on side (Fig. 18C). Anterior end of tignum narrow (Fig. 18B)	***Andersonoplatus macubaji***
–	Vaginal palpi separated on more than one third of their length (Fig. 22B). Posterior sclerotization of vaginal palpi slightly curved on side (Fig. 22B). Anterior end of tignum relatively wide (Fig. 22A)	***Andersonoplatus merida***
7	Sixth antennomere much smaller than seventh (Fig. 15C). Aedeagus in lateral view strongly curved, apical denticle (in ventral view) longer and better pronounced (Fig. 16A)	***Andersonoplatus lagunanegra***
–	Sixth antennomere as long as seventh (Fig. 34C). Aedeagus in lateral view nearly straight, apical denticle (in ventral view) shorter and less differentiated (Fig. 35A)	***Andersonoplatus saviniae***
8	Elytral interspaces flat	**9**
–	Elytral interspaces convex	**11**
9	Eyes small, with nearly 12 large ommatidia (Fig. 23B, D). Pronotum comparatively narrow (Fig. 23A). Apical denticle of male genitalia well developed, narrow (Fig. 24A)	***Andersonoplatus microoculus***
–	Eyes large, with more than 20, small ommatidia (Figs 7D, 12 D). Pronotum comparatively wide (Figs 7A, 12A). Apical denticle of male genitalia absent, poorly developed (Fig. 8A), or very wide (Fig. 13F)	**10**
10	Supracallinal sulci well developed, deep (Fig. 7D). Apex of median lobe of aedeagus bent ventrally in lateral view (Fig. 8A)	***Andersonoplatus castaneus***
–	Supracallinal sulci poorly developed, barely perceptible (Fig. 12D). Apex of median lobe of aedeagus straight in lateral view (Fig. 13F)	***Andersonoplatus jolyi***
11	Pronotal surface shiny, lacking punctures (Fig. 14A, B, E). Ventral side of median lobe of aedeagus flat with low longitudinal ridge apically (Fig. 14D)	***Andersonoplatus laculata***
–	Pronotal surface dull, covered with punctures (e.g., Fig. 2A). Ventral side of median lobe of aedeagus variously shaped but always without longitudinal ridge (e.g., Fig. 1F)	**12**
12	Pronotal surface uneven, covered with relatively large but poorly defined punctures (Fig. 5A). Median lobe of aedeagus ventrally with two ridges and deep groove between them (Fig. 6D)	***Andersonoplatus bechyneorum***
	Pronotal surface even, covered with moderately sized well defined punctures (e.g., Fig. 30A). Median lobe of aedeagus ventrally without ridges and deep grove between them (e.g., Fig. 1F)	**13**
13	Pronotal surface densely covered with well-defined punctures, diameter of which larger than distance between punctures (Fig. 30A). Second elytral stria reaching base of elytron (Fig. 30A)	***Andersonoplatus rosalesi***
–	Pronotal surface sparsely covered with variously defined punctures, diameter of which smaller than distance between punctures (e.g., Fig. 32A). Second elytral stria not reaching base of elytron (e.g., Fig. 32A)	**14**
14	Body yellow (Fig. 10A, B)	***Andersonoplatus flavus***
–	Body brown (Figs 1A, B, 32A, B)	15
15	Ventral side of median lobe with shallow longitudinal impression, bottom of which covered with transverse wrinkles (Fig. 2F). Spermathecal duct making relatively long loop (Fig. 2E)	***Andersonoplatus andersoni***
–	Ventral side of median lobe without longitudinal impression (Fig. 33A). Spermathecal duct making relatively short loop (Fig. 33E)	***Andersonoplatus sanare***

## Supplementary Material

XML Treatment for Andersonoplatus

XML Treatment for Andersonoplatus andersoni

XML Treatment for Andersonoplatus baru

XML Treatment for Andersonoplatus bechyneorum

XML Treatment for Andersonoplatus castaneus

XML Treatment for Andersonoplatus flavus

XML Treatment for Andersonoplatus jolyi

XML Treatment for Andersonoplatus laculata

XML Treatment for Andersonoplatus lagunanegra

XML Treatment for Andersonoplatus macubaji

XML Treatment for Andersonoplatus merga

XML Treatment for Andersonoplatus merida

XML Treatment for Andersonoplatus microoculus

XML Treatment for Andersonoplatus peck

XML Treatment for Andersonoplatus rosalesi

XML Treatment for Andersonoplatus sanare

XML Treatment for Andersonoplatus saviniae
